# Implementation of a real‐time, ultrasound‐guided prostate HDR brachytherapy program

**DOI:** 10.1002/acm2.13363

**Published:** 2021-07-26

**Authors:** Blake R. Smith, Sarah A. Strand, David Dunkerley, Ryan T. Flynn, Abigail E. Besemer, Jennifer D. Kos, Joseph M. Caster, Bonnie S. Wagner, Yusung Kim

**Affiliations:** ^1^ Department of Radiation Oncology University of Iowa Iowa City IA USA; ^2^ Department of Radiation Oncology University of Nebraska Medical Center Omaha NE USA

## Abstract

This work presents a comprehensive commissioning and workflow development process of a real‐time, ultrasound (US) image‐guided treatment planning system (TPS), a stepper and a US unit. To adequately benchmark the system, commissioning tasks were separated into (1) US imaging, (2) stepper mechanical, and (3) treatment planning aspects. Quality assurance US imaging measurements were performed following the AAPM TG‐128 and GEC‐ESTRO recommendations and consisted of benchmarking the spatial resolution, accuracy, and low‐contrast detectability. Mechanical tests were first used to benchmark the electronic encoders within the stepper and were later expanded to evaluate the needle free length calculation accuracy. Needle reconstruction accuracy was rigorously evaluated at the treatment planning level. The calibration length of each probe was redundantly checked between the calculated and measured needle free length, which was found to be within 1 mm for a variety of scenarios. Needle placement relative to a reference fiducial and coincidence of imaging coordinate origins were verified to within 1 mm in both sagittal and transverse imaging planes. The source strength was also calibrated within the interstitial needle and was found to be 1.14% lower than when measured in a plastic needle. Dose calculations in the TPS and secondary dose calculation software were benchmarked against manual TG‐43 calculations. Calculations among the three calculation methods agreed within 1% for all calculated points. Source positioning and dummy coincidence was tested following the recommendations of the TG‐40 report. Finally, the development of the clinical workflow, checklists, and planning objectives are discussed and included within this report. The commissioning of real‐time, US‐guided HDR prostate systems requires careful consideration among several facets including the image quality, dosimetric, and mechanical accuracy. The TPS relies on each of these components to develop and administer a treatment plan, and as such, should be carefully examined.

## 1 INTRODUCTION

Prostate high‐dose rate (HDR) brachytherapy using ^192^Ir is an established treatment modality that has recently been gaining popularity as a monotherapy treatment option for low‐ and intermediate‐risk prostate cancer and for boosting high‐risk prostate cancers. Its efficacy is recognized by the American Brachytherapy Society (ABS) and supported by a mature, multi‐institutional cohort of literature that has shown an increase in overall survival, disease control, and reduced toxicities.^1^, ^2^ In contrast to LDR brachytherapy, the intraoperative dose distribution is planned using a postneedle insertion image. As a result, there is a higher degree of confidence that the delivered dose distribution will match the planned dose distribution given the near temporal and spatial concurrence among imaging, planning, and delivery. The ability to optimize source dwell positions and dwell times among the inserted needles can result in superior healthy tissue sparing, target coverage, and potentially allow sub‐volume boosting of intraprostatic lesions, which has been hypothesized to further improve local control rates.^3^, ^4^ HDR prostate treatments have shown excellent 5‐year biochemical disease‐free survival when delivered in two to three fractions.^5^, ^6^, ^7^, ^8^ The quality and effectiveness of the HDR technique is likely associated with the precise capability to deliver the treatment plan.

HDR prostate brachytherapy includes a suite of imaging and treatment planning technologies. Historically, ultrasound (US) guidance has played an important role during LDR prostate seed implants (PSI) to confirm needle placement prior to implanting radioactive seeds in the prostate, as it is important to follow the preplanned needle placements as precisely as possible. However, other imaging modalities are generally preferred for treatment planning, such as MRI for enhanced soft‐tissue contrast or CT to help better visualize the seed locations. Unlike LDR where the entire treatment process extends several weeks between pre‐ and postplanning, an HDR treatment itself occurs over a period of minutes. The entire process of imaging, physician contouring, treatment planning, and delivery occurs within the span of several hours. As such, expedient imaging that preserves the treatment delivery geometry while also providing good prostate visualization is necessary to facilitate efficacious treatments. For HDR prostate treatments specifically, transrectal US provides real‐time imaging of the needle insertion and a prostate geometry definition that is identical between planning and delivery. These features can aid in improving the physician's ability to reproduce the preplanning needle distribution and assist the physicist to accurately reconstruct the needles. The latter can provide a large benefit dosimetrically if the treatments are optimized intra‐operatively. However, to fully exploit the dosimetric benefits requires an integrated system capable of intra‐operative imaging, reconstruction, planning, and delivery.

Intraoperative treatment planning systems have emerged that incorporate real‐time imaging into high‐dose rate (HDR) prostate brachytherapy, including the Oncentra Prostate system by Elekta (Stockholm, Sweden) and Vitesse by Varian (Palo Alto, CA). Both systems provide real‐time imaging via a transrectal US probe that is integrated within an intraoperative treatment planning system. While multiple reports have been published by the ABS, ESTRO, and the American Association of Physicists in Medicine (AAPM) to help facilitate the commissioning process, quality assurance procedures, and clinical practice of HDR brachytherapy,^1^, ^9^, ^10^, ^11^, ^12^, ^13^ dose prescription practices for common image‐guided treatment sites,^13^, ^14^, ^15^, ^16^, ^17^ brachytherapy treatment planning system dose calculations,^16^, ^18^ and brachytherapy US quality assurance,^19^, ^20^, ^21^, ^22^ a single task‐group or technical report document does not currently exist that explicitly sets forth a set of consensus guidelines to commission such an integrated system that combines US guidance with a delivery hardware of needles, templates, a stepper and stabilizer, and an intraoperative TPS for HDR prostate brachytherapy. In this regard, there are several shortcomings of the existing literature that fail to fully cover the scope and use of these real‐time, image‐guided HDR prostate treatment systems holistically in addition to their component‐wise functionality. For example, the AAPM task group (TG)‐40, TG‐56, and TG‐59 reports provide a code of practice and set of quality assurance guidelines for HDR afterloader units but do not include any recommendations on how imaging should be included or commissioned specifically for HDR procedures. The published AAPM TG‐128 report solely focuses on imaging QA of US probes used during HDR procedures but do not address the influence of image quality on the dosimetric planning accuracy. Similarly, the recent GEC‐ESTRO/ACROP recommendations also provide several image quality testing recommendations that complement the TG‐128 report and supplement mechanical and biplane calibration constancy tests, and thorough analyses have been performed validating US‐based HDR prostate planning with CT‐based planning.^23^ Similar commissioning works have also recently been published presenting the developed workflow, processes, and end‐to‐end testing used to commission other 3D image‐based treatment planning and delivery systems or applicators for cervical cancer brachytherapy and intracavitary breast electronic brachytherapy systems.^24^ However, similar reports are not currently present for US‐guided, intraoperative HDR prostate treatment systems. The fruition of these reports encompasses a comprehensive set of quality assurance tests for the US imaging and mechanical components but do not include any recommendations to evaluate the probe‐specific calibrations necessary to calculate the needle free lengths, which are critical parameters that rely on the culmination of imaging, mechanical, and treatment planning components.

The following report summarizes the acceptance and quality assurance (QA) measurements used to commission a real‐time, US‐guided HDR prostate brachytherapy system of Elekta System (Elekta AB, Inc.). These tasks are organized into imaging, mechanical, dosimetry, and end‐to‐end testing components. Additionally, this report summarizes the developed workflow, system of secondary checks, and clinical tools implemented at the UIHC for prostate HDR brachytherapy treatments. In this report, a specific Elekta system (Elekta AB, Inc.) was used as a vehicle to deliver the approaches and methodologies of acceptance and commissioning tests as well as developing a prostate HDR workflow.

## 2 METHODS AND MATERIALS

The tests and procedures discussed in this work were referenced from the available guidelines and publications to create a cohort of quality acceptance and commissioning protocols. The AAPM TG‐128 report^20^ was reference to assess the image quality and reconstruction accuracy of the brachytherapy US system. The AAPM TG‐43,^16^ TG‐56,^12^ and TG‐186 ^25^ reports were referenced to commission the treatment planning system, and mechanical quality assurance of the templates, needles, and stepper were evaluated following the recommendations from the TG‐40 report. Given the integrated nature of US imaging, a real‐time US‐guided, prostate HDR TPS (Oncentra Prostate, Elekta AB, Stockholm, Sweden) and hardware, supplemental tests that have been acknowledged by the GEC‐ESTRO^22^ were performed to benchmark the influence of artifacts, source calibration and output, mechanical and planning coordinate system coincidence, and validation of algorithmic approaches to monitor needle insertion depth. Specifically, this report introduces a set of additional commissioning tests that have not been reported in literature to evaluate the calibration of the probe's crystal‐to‐reference frame length, validate the TPS‐calculated needle free length both with and without encoder involvement, and benchmark the air‐Kerma strength degradation due to the presence of tissue inequivalent needles. A list of the commissioning tests, and tolerances where applicable, that were used during the commissioning the HDR prostate system is provided in Table 1. Within this report, many of the described procedures related to HDR brachytherapy commissioning experiences are detailed using product‐specific nomenclature from the manufacturer. However, the methods and techniques outlined in this report can serve as a general template to commission intra‐operative, image‐guided HDR brachytherapy systems regardless of the manufacturer.

**TABLE 1:** List of tests and their recommended tolerances assembled from numerous AAPM task groups, international reports, and specific investigations discussed in this work that were used to commission the imaging, mechanicals, and dosimetry components of a real‐time, US‐guided HDR prostate brachytherapy system

Test	Component	Tolerance	Report Reference
Grayscale visibility	Imaging	10%	TG‐128
Depth of penetration	Imaging	1 cm	TG‐128
Spatial resolution	Imaging	1 mm	TG‐128
Spatial accuracy	Imaging	2 mm (Axial) 3mm (Lateral)	TG‐128
Volume accuracy	Imaging	5%	TG‐128
Reference crystal‐to‐template calibration	Geometric	1 mm	This work
Manufacturer needle and catheter tolerance	Geometric	1mm	TG‐40, TG‐56
Source positioning	Geometric	1 mm	TG‐40, TG‐56, TG‐59
Needle template alignment	Geometric	3 mm	TG‐128
Stepper and encoder mechanical accuracy	Geometric	1 mm longitudinal 0.5° rotational	TG‐40
Offset calibration	TPS	1 mm	GEC‐ESTRO
Needle reconstruction	TPS	2 mm	GEC‐ESTRO
Needle free length	TPS	1 mm in an ideal image 2 mm with image artifacts	This work
Dose calculation	Dosimetry	2%	TG‐43, TG‐229
Air Kerma strength	Dosimetry	Evaluate, within 2% error	This work

**FIGURE 1:**
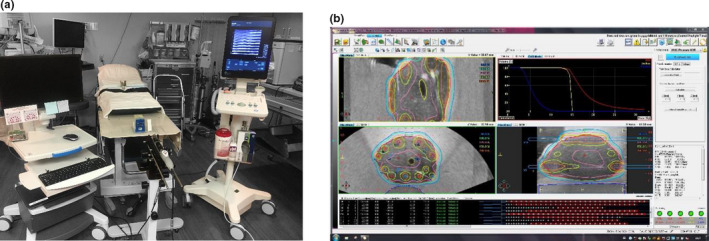
(a) The real‐time ultrasound (US) guided treatment planning system (OncentraProstate) that consists of the treatmenplanning system cart (left), the stepper and stabilizer holding the transrectal US probe (center), and the US unit (right). (b) An initial treatment planned on the TPS (OncentraProstate)

The setup for the real‐time US‐guided, prostate HDR system (Elekta AB, Stockholm, Sweden) is shown in Figure 1 and consists of the TPS (Oncentra Prostate), stepper and stabilizer, and the BK3000 US unit (BK Medical, Peabody, MA). The intra‐operative TPS (Oncentra Prostate) connects to both the BK3000 US unit and a Nucletron (Veenendaal, Netherlands) OncoSelect stepper system. During a procedure, the US image is monitored and stored within the TPS (Oncentra Prostate). Contouring and treatment planning are conventionally performed on the US image acquired with a BK medical E14CL4B trans‐rectal ultrasound probe, which serves as the primary image set for planning. Due to the dual crystal arrangement within the US probe, both transverse and sagittal views can be used interchangeably to monitor the treatment in real time or acquire volumetric scans. A unique feature of the TPS (Oncentra Prostate) is the ability to project the planned contours and virtual needles during the insertion of the needles into the prostate, which guides the physician and allows the physicist to reconstruct the needle geometry in real time. The projection of the electronic needle template and reconstruction of potential source positions are specific to the physical template and needle hardware, which where a recorded preset of the BK Medical 5F Trocar interstitial needles incorporated into the TPS (Oncentra Prostate) software.

### 2.1 Mechanical tests

#### 2.1.1 Needle insertion geometry

**FIGURE 2:**
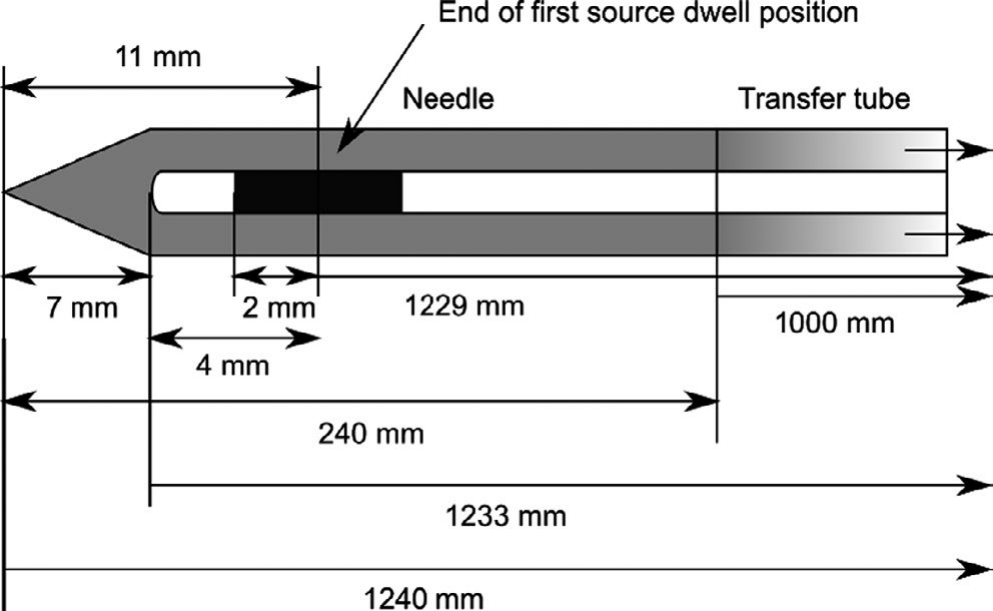
Schematic of the Trocar stainless steel interstitial needle attached to the 1000‐mm prostate catheters and an afterloading unit (Flexitron, Elekta AB.). Due to the location of the distal portion of the lumen 9 mm from the tip of the needle and a 4 mm length of the source encapsulation, the most distal dwell position is set 11 mm from the tip of the needle. There is 2‐mm safety margin between the source wire end tip and the inner lumen tip which is different design of Varian system (Varian Medical System, Inc.) Therefore, the expected index length including the catheter and source is 1229 mm

The needle reconstruction techniques used in this report reflects the methods from Zheng and Todor, which use a trans‐rectal ultrasound system to determine needle depth by monitoring the needle free length.^26^ For the TPS (Oncetra Prostate), the default parameters specify the nominal depths at which the needles are placed, the allowable dwell positions, and the user‐defined margin locality limit near the prostate clinical target volume (CTV). The geometry of the Trocar interstitial stainless steel needles (Elekta AB, Stockholm, Sweden), is shown in Figure 2. An important aspect of this needle is the maximum allowable dwell position between the tip of the needle and the center of the source, listed in Figure 2 as 11.0 mm and 6 mm for metal and plastic needles, respectively. The total length of the stainless‐steel needle is 240.0 mm and is a necessary parameter to verify the needle's reconstruction by benchmarking the calculated free length in the treatment planning system to the measured free length.

**FIGURE 3:**
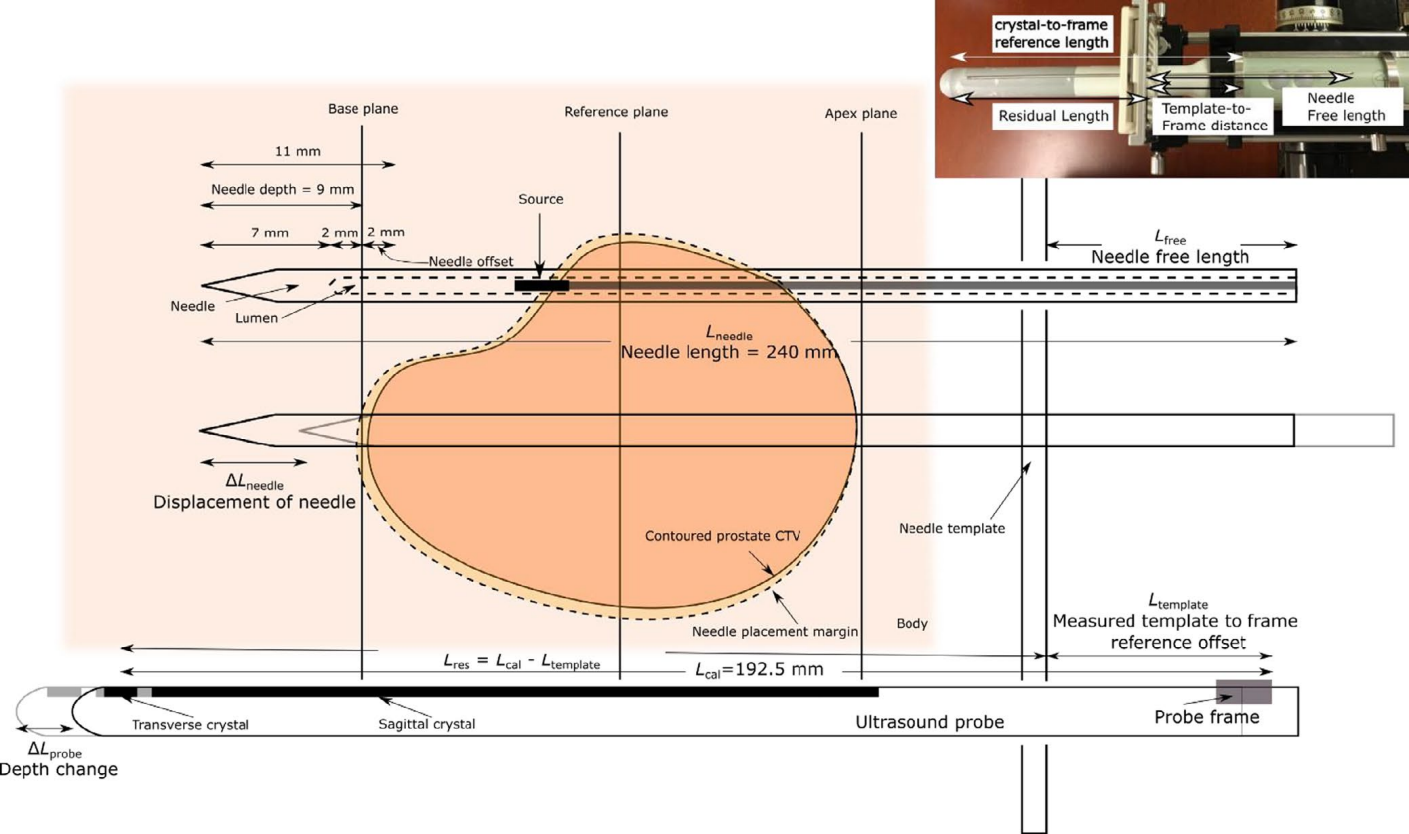
Initial needle insertion geometry used to calculate needle free length in the treatment planning system (Oncentra Prostate). A needle offset is set by the user, which systematically sets the needle depth for all virtual needles. Note that the allowed well positions rely on an independent parameter based on a marginal expansion of the prostate CTV contoured by the user

The calculation of free length, *L*_free_, is determined from the calibrated crystal‐to‐frame length, *L*_cal_, needle template‐to‐frame length, *L*_template_, the nominal length of the needle, *L*_needle_, and the amount of the needle that extends beyond the base plane when the needle is virtually placed in the TPS, $L_{\text{depth}}$. A distance of $\Delta L_{\text{needle}}$accounts for the distance the needle is offset from its nominal placement in the TPS. Figure 3 illustrates the needle and imaging geometry assumed by the TPS (OncentraProstate) to determine needle free length. The residual probe length,$L_{\text{res}}$, physically defines the depth of the base plane as an origin and is the value that is entered into the treatment planning system based on the measured template‐to‐frame length, $L_{\text{res}}$ = *L*_cal_ *– L*_template_. If the depth of the probe is changed by some distance, $\Delta L_{\text{probe}}$, without updating the base plane, the stepper encoder records the displacement to account for the apparent change in the needle's position relative to the measured residual length,$L_{\text{res}}$, by,

(1) $$L_{\text{free}}=L_{\text{needle}}‐L_{\text{res}}‐L_{\text{depth}}+\Delta L_{\text{needle}}+\Delta L_{\text{probe}}.$$

The addition of $\Delta L_{\text{probe}}$ is necessary as the base plane was set assuming a given *L*_template_. However, longitudinally displacing the probe changes *L*_template_, and thus $L_{\text{res}}$, by an amount of $\Delta L_{\text{probe}}$. To facilitate the calculations of this quantity during a brachytherapy procedure, an application was developed to calculate and maintain a historical account of template distance calibrations among HDR prostate treatments. The application tool is shown in Appendix A.

#### 2.1.2 Simulated source positioning and manufacturer tolerance

The Oncentra Prostate TPS relies on a representative model of the interstitial needle and catheter to facilitate needle reconstruction and set dwell positions. For this reason, it is recommended within the brachytherapy code of practice TG‐56 report^12^ and high dose‐rate brachytherapy treatment delivery TG‐59 report^10^ to rigorously check the geometric consistency and accuracy of the interstitial source and applicators as part of an institution's quality assurance program. Several factors outlined in the TG‐56 report^12^ were evaluated including the applicator dimensions, integrity, actual and simulated source positioning within the interstitial needles. The average and standard deviation in the length, outer diameter, and index length among 20 interstitial needles and transfer tubes were measured using a ruler micrometer (Mitutoyo, Aurora, IL), and a source simulator for the Flexitron Flexisource (Elekta AB), respectively.

#### 2.1.3 Stepper linear and rotational displacement

The real‐time, US‐guided TPS (Oncentra Prostate) relies on angular and longitudinal encoders to monitor the position of the probe to the patient anatomy relative to an initial reference point. These changes are monitored to properly determine needle free length, account for baseline shifts, and maintain geometric accuracy of the reconstructed longitudinal or sagittal scans. The precedent of mechanical QA has been established historically in the TG‐40 report for radiotherapy^11^ and is specifically addressed for US‐guided brachytherapy in the GEC‐ESTRO/ACROP recommendations for US imaging quality assurance for brachytherapy report.^22^ Following the GEC‐ESTRO/ACROP recommendations,^22^ the stepper was manually displaced between −1 mm and 10 mm longitudinally and referenced with a ruler. Likewise, a calibrated level was set on the cradle frame after an initial origin set, and the cradle was manually rotated between 0 and 90 degrees from the set origin. The measured displacements were then compared to the electronically recorded displacements using the ECRM motor testing programs and TPS software on the Oncentra Desktop.

#### 2.1.4 Template calibration

**FIGURE 4:**
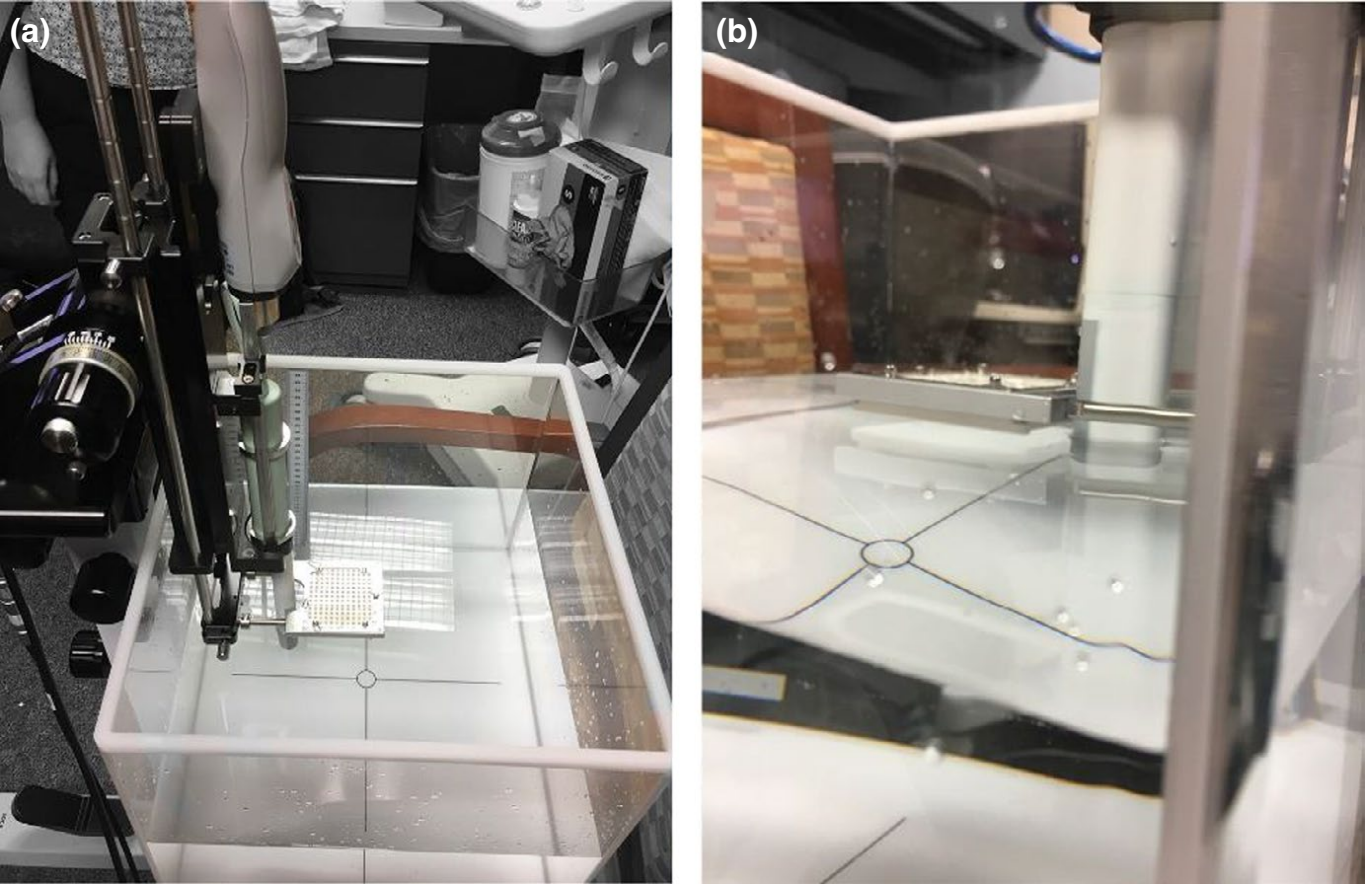
Ultrasound and stepper oriented vertically within a water phantom used to benchmark the coincidence between the physical and electronic templates

An electronic catheter template is available on the US system (BK3000) and the TPS (Oncentra Prostate) that serves as a reference of the available needle insertion points to the physician and physicist. Since the electronic template is specific to a particular physical template model, the TG‐128 report recommends verifying that the needle template is coincident and overlays on the electronic template before the first time a template is used and annually thereafter. A one‐dimensional scanning water tank was used to supply a large free‐scattering region surrounding the probe and template with enough distilled water to minimize reflection artifacts toward the exterior field of view as shown in Figure 4. The needle template (5f) was placed just above the water surface to minimize the potential deflection of an inserted needle before it registered at the plane of the transverse crystal. A single needle was inserted into the four corners and the central position of the template until it traversed the US’s transverse crystal's field of view. Upon registering the effective point scattering on the imaging system, the image set was frozen before any artifacts could blur the needle's detected position. The measuring tool on the US system was then used to measure the distance from the intended needle position, defined on the electronic template, to the center of the US‐detected position.

#### 2.1.5 Offset calibration

The transverse and sagittal crystals used to detect reflected US signals are longitudinally separated. This separation is calibrated by the manufacturer and incorporated into the TPS (Oncentra Prostate) to account for the relative shifts of the viewing planes when viewed in the software. Once the origin or baseline is set in the TPS (Oncentra Prostate), the location should remain in the precise location in space regardless of how the probe is translated thereafter. While there does not exist a commercial phantom to check this coincidence, the experimental methods proposed by Siebert et al.^15^ were used to benchmark depth of needle penetration and where adapted for this task.

**FIGURE 5:**
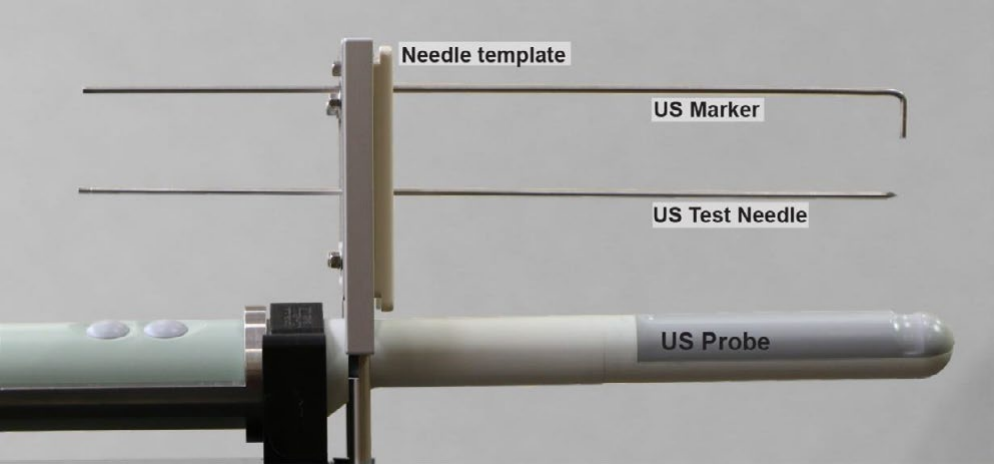
Setup of the ultrasound(US) depth marker fabricated from a stainless steel interstitial needle lengthwise across the US probe. A test needle was not used for offset calibration tests but was used later to evaluate depth of penetration accuracy

Spatial coincidence was established using a reference marker that was fabricated from an interstitial needle. As shown in Figure 5, the marker acts as a highly reflective point source in the transverse imaging plane. The needle tip was shaved flat in order to eliminate any signal anisotropy in the beam's‐eye‐view of the transverse crystal. A fixed marker depth, approximately 10 cm past the template, was secured with the needle parallel to the length of the US probe using the needle template and submerging the probe‐marker system vertically in a water tank.

#### 2.1.6 Needle depth accuracy

Absolute longitudinal needle positioning accuracy was performed in addition to recommended TG‐128 commissioning measurements. While this test is not discussed in the TG‐128 report, its importance is emphasized in the GEC‐ESTRO/ACROP recommendations for quality assurance of US imaging in brachytherapy.^22^ Under ideal phantom conditions, up to a 1.6 mm error has been report for US‐based needle reconstruction^26^ and up to 0.8 mm deviations in needle tip reconstruction.^21^ For this study, ground truth was defined using the US marker developed for the offset calibration tests. Unlike the offset calibration test, a test needle is used as a surrogate to evaluate the depth and free‐length accuracy of the TPS (Oncentra Prostate) and US system (BK3000). A derivative of the methods described by Siebert et al.^21^ were adopted for this work. Instead of inserting a needle until the user believes the needle's tip resides at the marker depth, the test needle was simply inserted approximately to the depth of the marker and physically measured from the surface of the needle template using a ruler, thus providing a known offset distance that was fixed with the needle template. The probe, needle, and marker were then submerged vertically in a water tank with enough space between the US marker and the distal edge of the water tank to minimize the contribution of reflected signals from the distal wall of the water tank.

#### 2.1.7 Crystal‐to‐frame distance calibration verification

**FIGURE 6:**
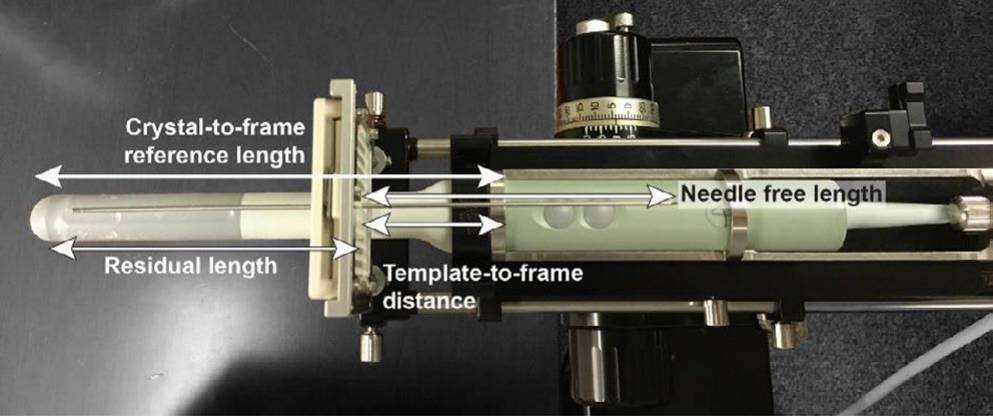
A constructed assembly of the probe mounted within the metallic cradle and inserted into the stepper. Free length calculations within the TPS (Oncentra Prostate) require a fixed geometry after the needle template frame and slide position are set and secured. During a procedure, the user measures the template‐to‐frame distance and enters the residual between the crystal‐to‐frame reference length and the measured template‐to‐distanced length

The algorithmic approach used by the TPS (Oncentra Prostate) to determine the needle free length requires that the distance from the center of the transverse crystal to the distal edge of the silver probe cradle ring that is most proximal to the template be calibrated and was 192.5 mm for the BK US E14CL4B probes used in this study. During a procedure, the template‐to‐frame distance is measured in order to set the origin of the treatment plan and determine the needle free length as Shown in Figure 6. No current recommendations exist between the AAPM, ESTRO, and ABS regarding the frequency that this parameter is checked. However, a nominal value should not be assumed for all probes due to manufacturing variability. Thus, each probe should be verified prior to its initial use clinically and annually thereafter.

**FIGURE 7:**
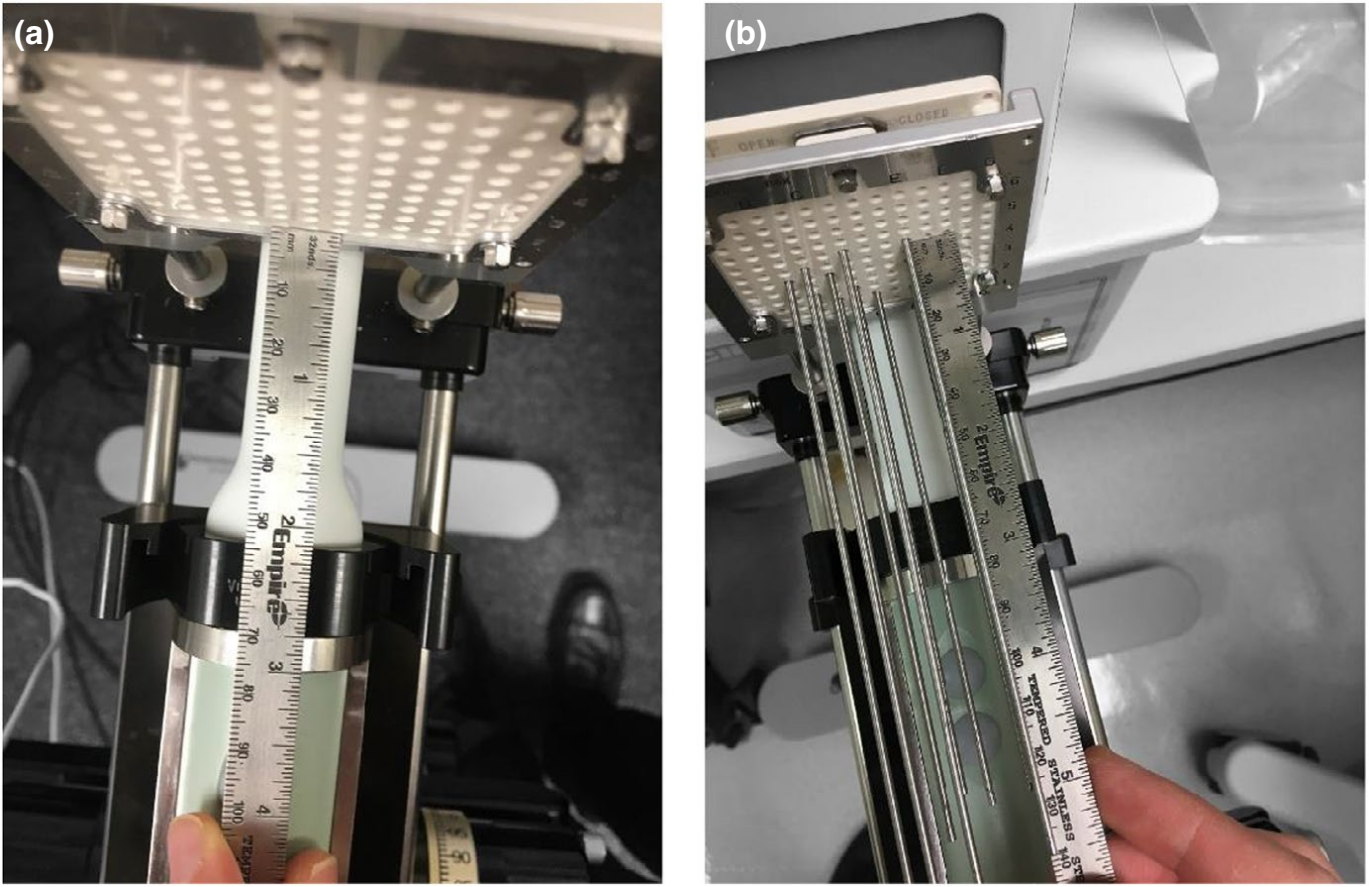
The experimental setup to check the crystal‐to‐frame calibration by performing a redundant check of needle free length compared to measurement. The initial template‐to‐frame (a) and subsequent needle free lengths (b) were measured using a ruler with 1‐mm precision markings

Variations of free length measurements were performed in a redundant fashion to verify the accuracy and consistency of the calibrated crystal‐to‐frame reference distance. In addition to the repeatability of measured free length, reproducibility should also be verified as this calibration is dependent on the setup of the probe inserted into a metallic cradle and placed on the stepper. The experimental setup for this validation test is shown in Figure 7. Prior to inserting a needle, the treatment planning system origin was set and the template‐to‐frame distance was measured. The residual from the nominal crystal‐to‐frame distance was entered into the treatment planning system. A sagittal scan was then acquired following the placement of the origin and insertion of a single test needle under ideal free scattering conditions.

### 2.2 Imaging tests

**FIGURE 8:**
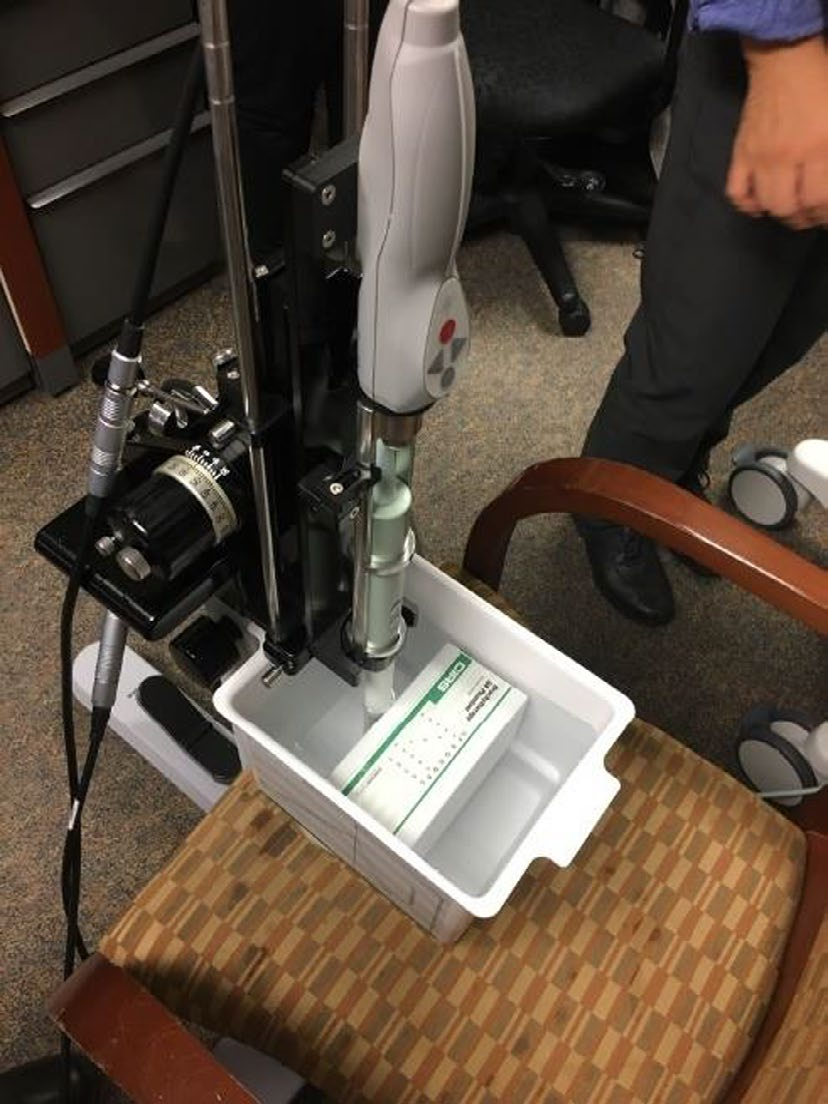
Spatial resolution and accuracy measurements were performed using the experimental setup where a CIRS 045B phantom is submerged in room‐temperature distilled water and set firmly against the ultrasound probed straddled in between the legs of an office chair where the water tank rests

A robust commissioning and quality assurance program for image‐guided, real‐time HDR prostate brachytherapy systems should include a comprehensive imaging component prior to evaluating the system holistically. Historically, the AAPM TG‐128 report has provided an excellent overview of suggested commissioning and quality assurance tests for these systems. However, the recommended quality control testing and workflow is demonstrated using an outdated CIRS phantom (Computerized Imaging Reference Systems, Inc.) that is no longer in production. While the majority of imaging tests performed in this report followed the recommendations from TG‐128 with a modern CIRS model 045B US phantom shown in Figure 8, a few modifications were made to benchmark the image quality of the transrectal US probes across the range of expected clinical frequency, which were 6, 9, and 12 MHz.

1. Low‐contrast detectability and visibility: The sensitivity of the system will reflect how deep into the patient a low‐contrast object can be detected and is largely governed by the signal‐to‐noise ratio of the system. Given that the full depth of the phantom was well visualized, a separate low‐contrast detectability test was performed in the open‐scattering environment shown in Figure 4. A plastic fiduciary marker was displaced radially from the probe until the detected contrast was notably degraded from the background signal.
2. Area and volume accuracy: The CIRS model 045B phantom contains three spherical objects with nominal volumes of 4 cm^3^, 9 cm^3^ and 20 cm^3^. Given the multi‐modality scanning features of the transrectal probes and Oncentra Prostate software, both axial and sagittal scans were used to quantify the volume of all three targets. The scans were then contoured by hand using the Pearl contouring tool in Oncentra Prostate. Area tests were not specifically performed it was assumed that volumetric accuracy infers areal accuracy and the CIRS phantom does not include object with calibrated, known areas.

### 2.3 Dosimetry tests

#### 2.3.1 Autoradiograph tests

**FIGURE 9:**
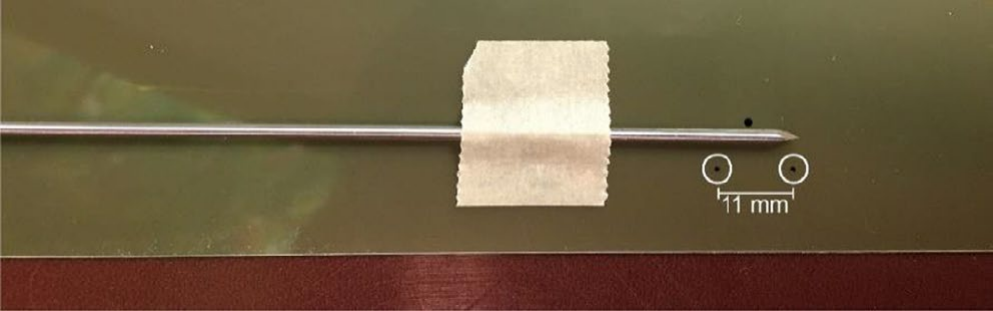
An image of the first irradiation setup to check the position of the most distal dwell position from the tip of the needle. Three fiduciary points were used to mark the tip of the needle and to supply a reference scale equal to the distance span of 11 mm between two points, which is the nominal distance between the tip of the needle and center of the most distal dwell position

The AAPM TG‐40, 56, and 59 reports recommend that source location, coincidence of dummy and active source be verified upon commissioning new applicators and yearly thereafter. This is particularly important for reusable interstitial needles as the manufacturing and resulting source positioning must be both accurate and precise as a single needle model is assumed among multiple applicators. A series of autoradiographs were acquired to check the source dwell positions relative to one another and the tip of the interstitial needle. HDR deliveries were created to administer a sequence of dwell positions along a single catheter. Two deliveries were anti‐parallel with a single, 10‐s dwell position at the distal‐ most dwell position and the third delivery consisted of three 5 s dwell positions spaced 1 cm apart with the first dwell position also located at the distal‐most dwell position. The needle was taped on top of a piece of Gafchromic EBT3 film (Ashland, Bridgewater, NJ) as shown in Figure 9. Markings were made in permanent marker to specify the location of the needle tip and to benchmark the image distance scaling.

#### 2.3.2 Air‐Kerma strength determination

The air‐Kerma strength parameter that is updated within the TPS (Oncentra Prostate) reflects the source strength measured under the conditions of the calibration at the time of the source exchange. Any source or applicator differences from the initial calibration setup could lead to discrepancies in the dose rate. Therefore, the impact of the stainless steel interstitial needles on the resulting air Kerma strength was quantified relative to the measured air‐Kerma strength within a plastic catheter, which is the conventional applicator that is used to measure the source strength at the time of a source exchange.

The air‐Kerma strength of the ^192^IrHDR source was measured using the Flexitron (Elekta AB, Inc.) afterloader and current Flexisource ^192^Ir source (Elekta AB, Inc.) using the conventional plastic catheter and interstitial needle. Measurements were carried out using an HDR 1000 Plus well‐type ionization chamber (Standard Imaging, Inc.) and a CDX‐2000B electrometer (Standard Imaging, Inc.). Sweet spot determinations were initially carried out for each catheter by displacing the source position within the chamber to map out the sensitivity profile. Temperature, pressure, electrometer, and calibration coefficients were applied to the final current readings to determine the measured air‐Kerma strength of the source delivered among the two applicators.

#### 2.3.3 Dose calculations

**FIGURE 10:**
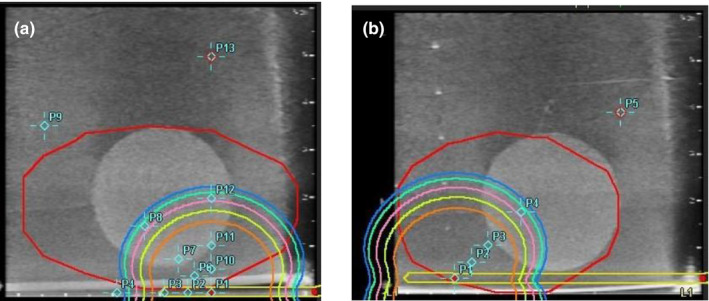
Screenshots of the approximated single‐source dwell position calculations for all locations between 0 to 90 (a) and 135 (b). A pseudo PTV contour, shown in red, was generated to allow dwell positions near a specified region of the scan. The two dwell positions, shown as red dots, where manually weighted based on dwell time so that the dominate position was five orders of magnitude larger than the second dwell spot and space geometrically far apart

A three‐part dose calculation comparison was used to validate the ^192^Ir source model (Flexisource, Elekta AB., Inc.) and commission the TPS (Oncentra Prostate) and an independent dose calculation software (RadCalc, LAP GmbH Laser Applications, Austin, TX) redundantly with a manual dose calculation following the TG‐43 formalism.^16^, ^18^ Calculations of the source geometry functions were performed using the manufacturer source dimensions, which included a 3.5‐ mm active source length. Radial and anisotropy functions were evaluated from the consensus data set forth by the AAPM and ESTRO,^18^ which includes the Monte Carlo and experimental results from Granero et al.^27^ and Taylors and Rogers.^28^ A total of 16 dose points were calculated about a virtual source position using a surrogate US scan to calculate dose points from an intended, single dwell position. Two source locations were necessary to calculate entirely around the source for the desired radially distances due to the limited field of view in the TPS (Oncentra Prostate) shown in Figure 10.

In addition to benchmarking the consistency of the TG‐43 source models, the secondary dose engine RadCalc was also commissioned to serve as an independent dose calculation verification for the HDR prostate treatment plans, which has been a primary focus of development for both LDR, HDR, and PDR modalities.^29^ A set of five end‐to‐end tests were performed using the tissue‐equivalent CIRS 053S ultrasound prostate phantom. Included within these tests were a comparison between the secondary dose calculation software and the treatment planning system. A set of six points were selected throughout the treatment volume including two points near the apex, base, and within the central portion of the prostate. At least one of the points was placed within the urethra. The final dose calculation from the secondary dose calculation software was compared against the treatment planning dose determination for each of the six points.

### 2.4 Workflow development and end‐to‐end tests

A step‐by‐step treatment procedure and sequence of secondary checks were designed to facilitate an expedient treatment delivery while also minimizing treatment delivery errors. As there does not exist any current recommendations regarding the design of an US‐guided, real‐time HDR prostate treatment program, the general recommendations from the AAPM TG‐59 report^10^ and consultations among multiple clinics with active US‐guided, real‐time HDR prostate programs that included the University of Nebraska Medical Center, University of California Los Angeles, and the University of Wisconsin Madison were used to design a robust and comprehensive treatment procedure. These tasks include the logistics of equipment and personnel in addition to a clear sequence of events organized in a manner to promote redundant checks. The procedural tasks were separated among the following healthcare professionals: the radiation oncologist (RO), planning (i.e., primary) physicist (P), secondary physicist (2P), registered brachytherapy nurse (N), and the radiation therapist (RTT). The full workflow is included as a supplemental document to this work. A series of redundant checks were also incorporated, including two supplemental secondary check scripts that were designed to validate the needle configuration and monitor the dose‐volume histogram OAR constraints and CTV coverage. Multiple test runs were performed to time and validate the entire workflow process.

## 3 RESULTS

### 3.1 Simulated source positioning and manufacturing tolerance

A set of simulated source position measurements were completed for all HDR prostate catheters among several Trocar stainless steel interstitial needles. The nominal reported catheter and needle lengths from the manufacturer were 1000 mm and 240 mm, respectively. However, the distance between the tip of the Trocar needle to the most distal dwell position as shown in Figure 2 is 11 mm. The average and standard deviation in the length, outer diameter, and index length among 20 interstitial needles and transfer tubes are listed in Table 2.

**TABLE 2:** Summary of basic geometric acceptance checks among several needles and transfer tubes

Parameter	Nominal	Measured (mm)	
	Value (mm)	Mean	σ (k = 1)
Needle length	240	240	0
Needle outer diameter	1.9000	1.884	0.004
Index length^a^	1229.0	1229.5	0.4

^a^ Index length defined at the depth of the source center position at its most distal dwell position after traversing the transfer tube and Trocar interstitial needle.

### 3.2 Stepper linear and rotational accuracy

The longitudinal displacements recorded from the stepper encoder and presented on the Oncetra Prostate TPS were found to be within 0.2 mm of the measured displacements for the range of translations studied in this work. Measured and recorded angular displacements within the TPS agreed to within 0.5 degrees.

### 3.3 Autoradiograph tests

**FIGURE 11:**
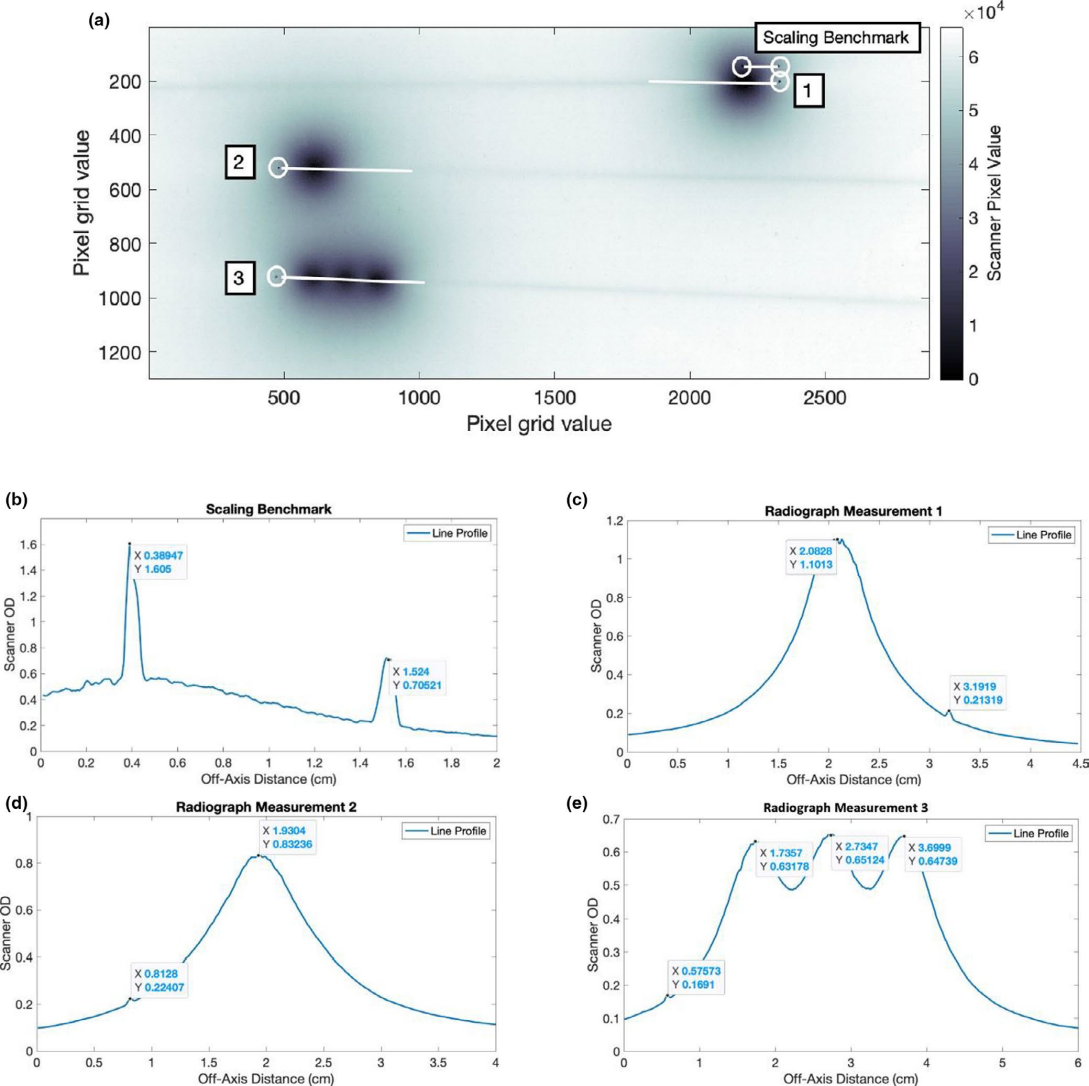
(a) The raw scan from the source autoradiograph measurements that are labeled accordingly to the subsequent plots in b‐e. Line profile measurements using Gafchromic EBT3 film of the (b) image scaling test, (c) first single dwell position, (d) second single dwell position, and (e) sequence of three equally spaced dwell positions. Dwell position is inferred toward the maximum in the center of each Gaussian‐like source profile. Leptokurtic profiles are indications of referencing markings placed with permanent marker

Post‐irradiation scans were acquired with an EPSON 11000X flatbed scanner (Dell) and analyzed in MATLAB (R2019a) (MathWorks). The scanner signal was converted to raw optical density from the maximum red color channel pixel intensity value within a 64‐bit image. As illustrated in Figure 11, three line profiles were used to benchmark the scaling and measure the relative source spacing among other source dwell positions or the tip of the needle. Source position was inferred using the center of line profile maximum calculated from the optical density values and off‐axis distance. Distance scaling was checked using a basic scaling examination test between two points separated by a known physical distance. The results from the Gafchromic EBT3 film measurements are listed in Table 3. The difference between the marked and measured source position was less than 1.0 mm.

**TABLE 3:** Results from the source position radiograph test

Fiducial test	Maxima position (cm)			Displacements (cm)		
	Pos 1	Pos 2	Pos 3	Mark & Pos 1	Pos 1 &2	Pos 3 &4
Scaling	0.389	1.524		1.135		
Radiograph 1	3.919	2.083		1.109		
Radiograph 2	0.813	1.930		1.118		
Radiograph 3	0.576	1.736	2.736	1.160	0.999	0.965

The scaling benchmark had two marks spaced apart a known distance of 1.1 cm. Additionally, the nominal source‐to‐fiducial (i.e., needle tip position mark) was also 1.1 cm. Additional source dwell positions were displaced 1.0 cm apart.

### 3.4 Template calibration accuracy

Five regions were investigated that included the four corners and the central position of the template. The displacement measurements from their nominal location presented on the US system's electronic needle template are listed in Table 4.

**TABLE 4:** Template alignment differences (in mm) between measured needle positions among two different US probes and physical needle templates

Measurement point	Frequency (MHz)	Probe 1		Probe 2	
		Template #1	Template #2	Template #1	Template #2
a6	6	2.83	2.17	2.39	2.96
f6	6	2.75	2.19	2.91	2.65
a1.5	6	0.13	1.66	1.13	1.60
f1.5	6	1.90	0.53	0.80	1.86
D3.5	6	2.64	1.22	1.51	2.18
a6	9	2.39	1.97	2.12	2.39
f6	9	1.74	2.21	1.93	2.09
a1.5	9	1.31	2.25	0.26	0.53
f1.5	9	0.66	0.85	1.59	1.22
D3.5	9	1.78	1.48	1.88	2.26
a6	12	2.65	2.63	2.05	2.00
f6	12	2.02	2.14	1.78	1.97
a1.5	12	0.77	0.71	1.07	1.74
f1.5	12	1.72	0.00	1.46	2.07
D3.5	12	2.46	2.16	1.72	1.78

Differences represent the magnitude of positional error determined from the needle positions detected on a transverse US image and the electronic template location of needles.

### 3.5 Crystal‐to‐template distance calibration verification

Software tools within the TPS (Oncentra Prostate) were then used to reconstruct the needle using a preprogrammed model of the interstitial trocar, stainless steel needle, and the resulting free length calculated by the TPS (Oncentra Prostate) were compared against the measured free length. This process was repeated by re‐inserting the needle to different depths and by changing the probe depth with different origin locations. The results from this study are listed in Table 5.

**TABLE 5:** Parameters and results following the free length analysis used to test the crystal‐to‐frame distance calibration

Template	Distance to(mm)		∆ (mm)	Needle Free Length (mm)		
	Frame	Crystal		Measured	TPS	Difference
1	78.0	114.5	0	134.0	233.43	0.57
2	63.5	129.0	0	125.0	125.08	−0.08
1	70	122.5	15.5	124.0	123.5	0.50
1	56	136.5	21.9	116.5	115.5	0.90

The distance, D, between the template and frame was varied with different origin sets. The residual of from the calibration distance (reported as 192:5mm) and the measured template‐to‐frame distance was monitored by hand as it is treated in the software while also accounting for longitudinal shifts of the ultrasound probe, ∆.

### 3.6 Offset calibration

**FIGURE 12:**
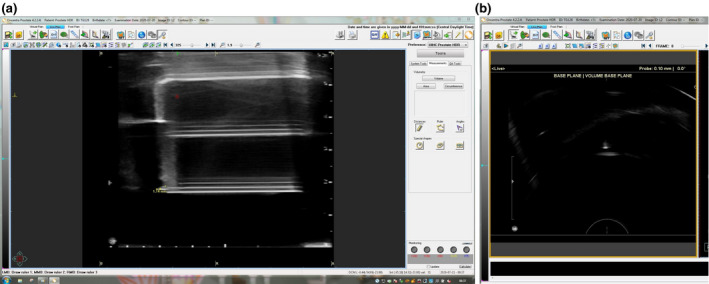
Sagittal (left) and transverse (right) ultrasound images of the ultrasound marker. The baseplane was set in the sagittal view within the center of the marker directed toward the ultrasound probe. Note that the probe depth is the depth of the transverse crystal, which is beyond where the baseplane was set. Upon setting the baseplane, and retracting the ultrasound probe, the depth of the brightest signal from the marker in the transverse plane, shown in the upper right‐hand corner of (right), was compared to the base plane position set in the sagittal view, which was 0.00 mm by definition

The scanner was initially set in the sagittal view and manually rotated to find the plane of the US marker as shown in Figure 12. Using the TPS (Oncentra Prostate), a new base plane was placed along the center of the marker directed toward the US probe. Once the base plane was set and a new scan was acquired, the US system was switched to transverse mode and the probe was manually retracted with the stepper to the longitudinal position that resulted in the US marker's greatest US signal. The plane depth listed in the TPS (Oncentra Prostate) was compared to the expected base plane position. Up to 0.1‐mm difference was observed between the sagittal and transverse base planes.

### 3.7 Needle depth accuracy

**FIGURE 13:**
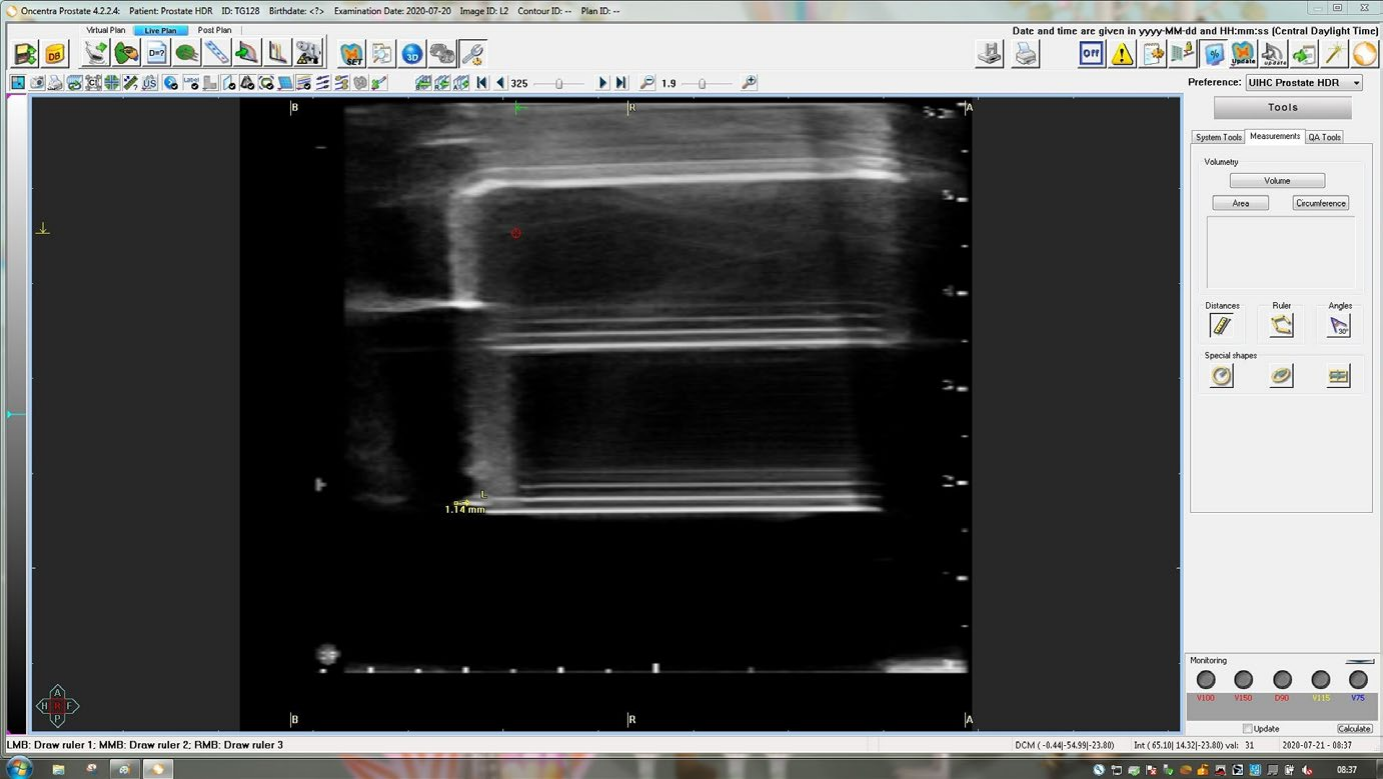
Needle tip displacement measurement from a reconstructed needle in the TPS (Oncentra Prostate) to the ultrasound marker imaged from a sagittal scan. Displacement was measured from the discernible tip of the needle to the center of the marker

**FIGURE 14:**
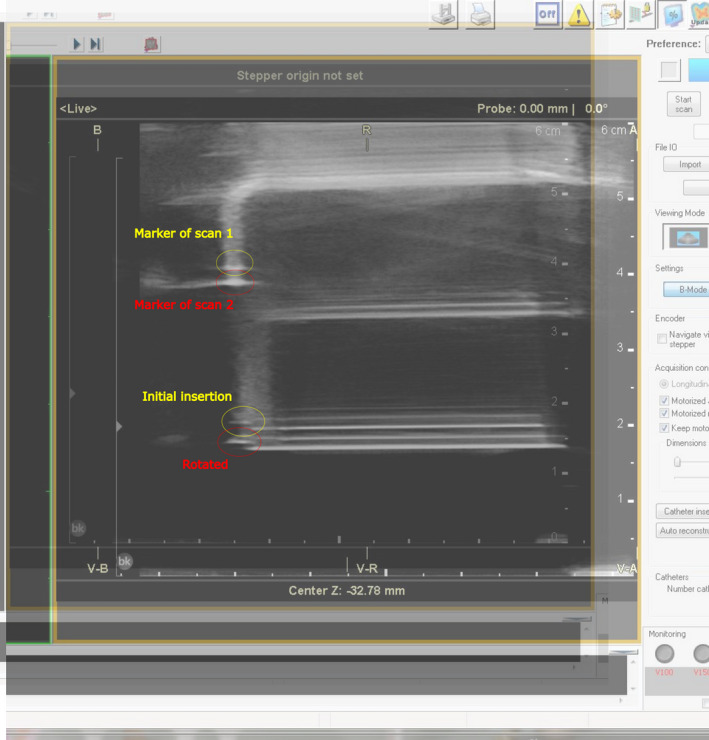
Sagittal ultrasound (US) images of the in‐house machined US maker and an interstitial needle. Two images are registered in depth to the center of the US marker but displaced vertically to showcase the differences in the needle's measured position from the US due to the needle being rotated

A sagittal scan was then acquired of the needle and US marker, and the difference in depth between the marker and needle tip was quantified using the measuring tools available in the TPS (Oncentra Prostate). Figure 13 presents one of the test cases from the results listed in Table 6 that was analyzed in the TPS (Oncentra Prostate). These results show that millimeter longitudinal position accuracy is achievable under ideal conditions. Under these same conditions, inter‐user variability was minimal as each run listed in Table 6 was performed by a different individual and agreement was within 1 mm. Repeated scans were acquired with the needle rotated from its initial alignment to the US probe. A superposition of two images is shown in Figure 14 where the scans have been registered longitudinally at the center of the ultrasound marker but displaced vertically to illustrate the change in depth of the needle's tip. Marginal differences, less than 1 mm, in the needle's depth were observed due to the anisotropic scattering surface of the trocar interstitial needle, which is consistent with the published data from Siebert et al.^21^

**TABLE 6:** Needle depth accuracy measurements between the reconstructed needle in the TPS (Oncentra Prostate) and the center of the ultrasound marker compared to the known, measured offset

Object	Free length (mm)	Difference (mm) From TPS	Measured (mm)	
			Depth	Difference
US Marker	10	‐‐	126	‐‐
Needle Run 1	10	1.14	127	1
Needle Run 2	10	1.04	127	1

### 3.8 Dose calculation algorithm

A comparison between the hand‐calculated and treatment‐planning calculations from the TPS (Oncentra Prostate) are listed in Table 7. Excellent agreement was observed between the hand and TPS‐calculate dose points, which differed by tenths of a percent for all points and is well within the calculation tolerance of 2% recommended in the TG‐43^16^ and TG‐229^18^ reports. Excellent agreement was observed among the tested cases. The largest deviation between RadCalc and the Oncentra Prostate treatment planning system for all dose points among the dry‐run treatment plans was 0.1%, which was well within the 2% acceptance criteria recommended in the TG‐43 report.

**TABLE 7:** Comparison between manual TG43 Calculations, TPS‐calculated doses in Oncentra Prostate (TPS), and RadCalc secondary calculation checks to points surrounding a Flexisource source

Angle (degrees)	Radii (cm)	Point doses (Gy)			Percent difference (%)		
		Manual	TPS	RadCalc	Manual	Manual	TPS
					TPS	RadCalc	RadCalc
0	0.50	173.08	173.45	173.45	−0.22	−0.22	0.00
0	1.00	37.08	37.11	37.06	−0.09	0.07	0.15
0	2.00	9.22	9.23	9.25	−0.07	−0.28	−0.21
0	5.00	1.62	1.62	1.62	−0.04	−0.30	−0.26
45	0.50	231.80	231.78	231.68	0.01	0.05	0.04
45	1.00	56.31	56.39	56.43	−0.13	−0.21	−0.08
45	2.00	14.07	14.09	14.10	−0.11	−0.20	−0.09
45	5.00	2.25	2.25	2.26	−0.02	−0.11	−0.09
90	0.50	223.38	223.75	223.72	−0.16	−0.15	0.01
90	1.00	57.68	57.71	57.68	−0.06	−0.01	0.05
90	2.00	14.58	14.59	14.59	−0.06	−0.05	0.02
90	5.00	2.33	2.33	2.33	−0.04	−0.04	0.00
135	0.50	231.80	231.00	231.03	0.35	0.33	−0.01
135	1.00	56.43	56.40	56.38	0.06	0.09	0.03
135	2.00	14.08	14.08	14.08	0.00	0.00	0.00
135	5.00	2.25	2.25	2.25	−0.08	−0.08	0.00

A nominal dwell time of 999 s was assumed in addition to a dose‐rate conversion factor of 1.113 cGy/h/U.

### 3.9 *Air*‐*Kerma strength determination*

Single‐spot dwell irradiations were delivered at the sweet spots unique for each applicator, which were 242 mm and 189 mm for the plastic catheter and interstitial needles, respectively. Due to the 11‐mm space between the tip of the Trocar needle and the center of the most distal dwell position, the well‐chamber sensitivity curve and sweet spot location of the needle appears shifted in comparison to the standard catheter. The air‐Kerma strength for the current 192Ir source (Flexisource, Elekta AB) was reduced by 1.12% using the stainless steel needle.

### 3.10 Developed workflow

**FIGURE 15:**
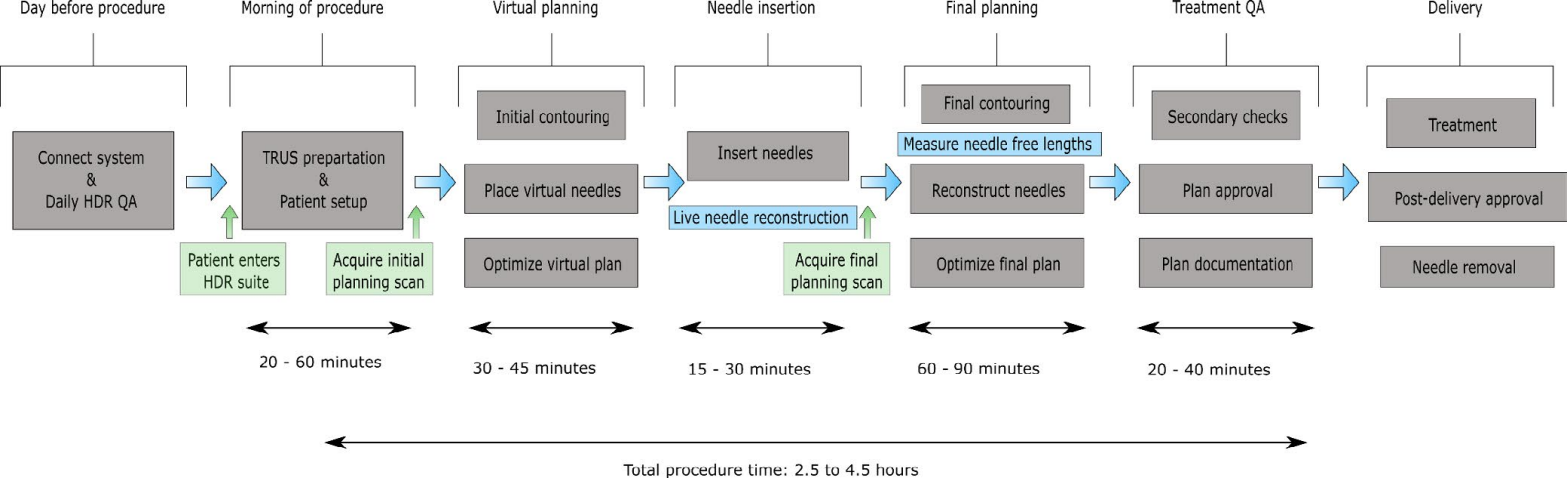
Flowchart of the HDR prostate treatment procedure using real‐time US imaging and an intraoperative TPS. The entire procedure is compartmentalized in seven stages illustrated among the rows. Primary tasks are categorized in gray boxes with important subtasks that may be completed simultaneously shown in blue boxes. Green boxes indicate important miles stones during the procedure between certain stages. Time estimates are provided in a realistic range of expectations barring serious setbacks or unforeseen complications with either the patient or the treatment system

The developed workflow was segmented into six components and three sets of check lists. An expected timeline of the full clinical procedure is shown in Figure 15. Copies of the checklists are included in the supplemental procedure workflow document, and illustrations of the secondary check scripts are also included: the templates of the spreadsheets used to record the needle placement and free length measurements (Appendix B) and monitor the DVH treatment planning goals and constraints (Appendix C).

**FIGURE 16:**
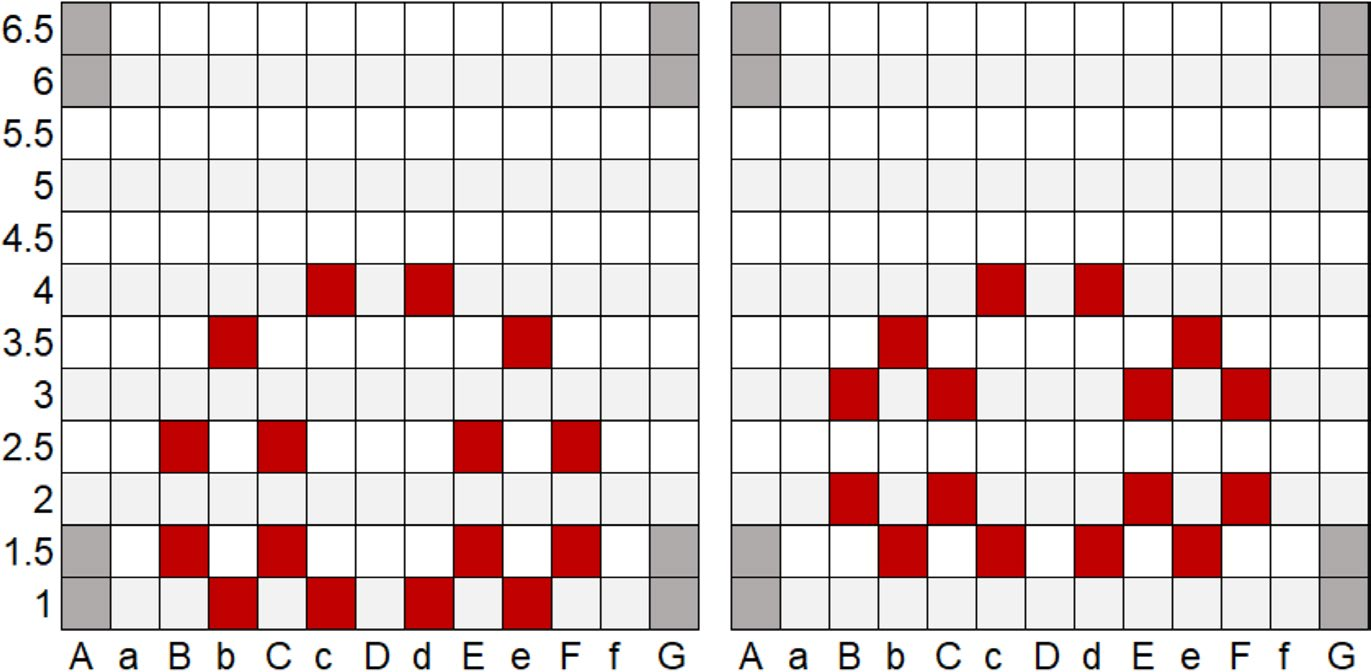
Standard needle pattern for a 16‐needle treatment plan that can be used for larger (left) and smaller (right) prostates. Needle insertion points are highlighted in red to visualization schematic for 6F physical templates (BK and Elekta AB)

During workflow development, the preparation list prior to treatment day was developed that includes: all equipment should be cleaned and any additional equipment that is not currently available should be ordered. The sterilization and cleaning protocol institutionally developed is included as a supplemental document to this manuscript. The stepper and stabilizer are assembled and placed in the HDR suite along with the treatment planning computer cart. All cables connecting the stepper, US probe, and computer system should be connected, ensuring proper electronic communication among these devices and proper presets. In addition, the preparation list on the day of treatment was also developed, including that a transrectal US balloon will be used for HDR prostate procedures in order to improve the US image quality and physically displace the prostate anteriorly, centering it within the needle template. The US probe and balloon should be assembled several minutes before the procedure and arranged vertically within the stepper to alleviate any residual bubbles that may remain on the probe. A planning physicist will review the hardware setup and check that the field depth is detected in the software, the electronic needle templates match between the US and TPS (Oncentra Prostate) and create a new patient file in the database. A nurse will simultaneously set up a sterile table, which consists of the interstitial needles, obturators, stabilization needles, and the physical needle template. Through multiple dry‐runs with physicians, nurses, radiation therapy technicians (RTTs), and medical physicists, the detail steps of US probe insertion, US imaging, initial virtual treatment planning, needle insertion, and final planning on US imaging with needle insertions were determined and documented. During workflow development, institutional standard needle loading patterns were developed based upon the recommendation of AAPM brachytherapy school,^30^ which are shown below in Figure 16. The developed institutional standard loading needle pattern utilizes 16 needles, which additional or fewer needles may be used. However, the ABS consensus guideline^1^ and RTOG0924 report^31^ recommends at least 14 needles to minimize hotspots and RTOG 0924 report^31^ recommends no more than 20 needles to improve coverage robustness.

#### 3.10.1 Developing institutional prostate HDR plan evaluation parameters

The institutional plan evaluation metrics were developed based on the ABS consensus guidelines^1^ as well as the culmination of clinical experiences of the Sunnybrook Odette Cancer Centre, the UCLA School of Medicine Department of Radiation Oncology, the University of Nebraska Medical Center (UNMC), and the Centre Hospitalier de L’Universite de Montereal. Institutional prescription scheme of monotherapy HDR treatments will be given in two fractions with each fraction prescribing 13.5 Gy to the CTV and boost HDR treatments consisting of a single 15 Gy fraction. These metrics were adopted from RTOG reports 0924^31^ and 0815.^32^ While there is a lack of consensus on the specific coverage of either a CTV or PTV, the institutional plan evaluation DVH parameters were to have a CTV V100% >95%, CTV V150% <35%, the dose delivered to 90% of the CTV volume (CTV D90%) between 100% and 115%, urethra V115% <5% and the maximum fraction of the prescribed dose experienced within 2 cubic centimeters of the rectum (D2cc) <70%. A plan DVH evaluation tool in an Excel format (Microsoft Corp.) was developed and presented in Appendix C.

#### 3.10.2 Developing checklists

During prostate HDR workflow development, four checklists were developed for a planning physicist, a second check physicist, an RTT, and a nurse. For a nurse checklist, it lists out the items that need to be prepared and get ready in the operation room. The planning physicist's checklist focuses on the treatment computer console and hardware communication throughout the treatment. The second physicist is primarily responsible for redundant checks on the patient setup and treatment planning throughout the procedure. The RTTs serves as an additional redundant check to verify patient setup, the completion of pre‐ and posttreatment documentation, and manually records the needle insertion configuration and measurements acquired by either the primary or secondary physicist. The developed checklists were iteratively updated throughout commissioning and two full HDR staff dry‐runs.

## 4 DISCUSSION

An HDR dose distribution is particularly sensitive to the needle reconstruction accuracy. As much as a 3% error can occur within the high‐dose, low‐gradient regions for every 1 mm an actual dwell position differs from the planned position^11^ and as much as 274% for a point located 2 mm distally from the tip of a needle.^21^ The advent of real‐time imaging and needle reconstruction temporally close to the actual delivery aims to improve these uncertainties, but accurate delineation of the needles and their correct reconstruction is still a factor that must be considered, especially for HDR treatments. While the primary scope of these materials focuses on the establishment of an US‐guided, intraoperative prostate HDR treatment system, failure mode error analysis (FMEA) is a necessary component to consider but was intentional omitted from this work. A complete and thorough FMEA requires a cohort of experiences acquired from multiple patient treatments and is an available product once the program has matured. As such, a robust FMEA may be outside the scope of the initial commissioning and is the ongoing focus of future work.

### 4.1 Commissioning measurements

The commissioning of the real‐time, US‐guided prostate HDR system was categorized into three parts: the treatment planning system, the US guidance system, and hardware system. However, each of these components are critically interconnected among each other, especially given that the technology is intended to be used as an intra‐operative system. Therefore, it is important to identify the source of any potential failure of the system holistically in addition to each component separately. For this reason, the TG‐128 task group recommendations^20^ were followed to commission the US system and probe, TG‐43^16^ evaluations were performed to check the calculation accuracy of the TPS (Oncentra Prostate), and TG‐40^11^ as well as TG‐56^12^ acceptance testing of the new applicators were completed. Finally, end‐to‐end tests are performed to verify the deliverability of the entire procedure.

While most of the recommendations from this report focus on the US system, these recommendations were expanded to include the TPS (Oncentra Prostate) as necessary. System‐specific checks were performed to verify the origin coincidence of the scanning crystals in the TPS (Oncentra Prostate), the crystal‐to‐frame calibration distance of the probe assumed by the TPS (Oncentra Prostate), the electronic accuracy of the stepper motor encoders, and the longitudinal needle positioning accuracy. The results of this work's needle positioning verification and inter‐contouring variability listed in Table 6 closely resemble the results from Siebert et al.,^21^, ^22^ which demonstrated a needle position accuracy relative to a fiduciary mark of 0.1 mm and 1.8 mm depending on the US manufacturer and model and longitudinal scanning direction and standard deviations ranging between 0.2 mm and 0.8 mm.

Absolute needle positioning requires that an external calibration distance be known such as the center of the transverse crystal to the portion of the US probe's cradle, which is used in the TPS (Oncentra Prostate) to calculate needle free length. As demonstrated in this work, the calibration and consistency of the US system, stepper motors and encoders, and the TPS (Oncentra Prostate) can be evaluated based on the agreement between the measured and calculated free length. The results listed in Table 5 demonstrate the redundant determination of needle free length by measurement and calculation, which was evaluated by hand as well as within the TPS. (Oncentra Prostate). While differences between the measured and calculated free lengths exist, these differences appear on the order of the precision of the needle contouring itself and constitutes the dominant limit to the accuracy of the crystal‐to‐frame calibration.

Dosimetric calculation accuracy was separated into calculation and output verification. The TG‐43 parameters used to model the source were checked based on the source model specified within the TPS (Oncentra Prostate) and by a comparison of calculated TG43 point doses. As presented Table 7, excellent agreement was found between the TPS (Oncentra Prostate), the secondary dose calculation engine (RadCalc), and by the manual TG‐43 calculations. Additional measures have also been suggested in addition to independent secondary dose checks, such as the use of nomograms as an independent quality assurance measure to benchmark the total delivered air‐Kerma strength of the treatment.^33^ However, these calculations should not be considered sufficient to fully verify the dosimetry. Using a seven‐distance technique, the air‐Kerma strength measured using an Exradin A3 ionization chamber is transferred to a standard well chamber that is later used to transfer the air‐Kerma strength calibration to a customer well chamber using a redundant, replacement technique.^34^ Any deviation from the specific experimental conditions of this calibration may affect either the source's air‐Kerma strength or the measured air‐Kerma strength overlooked by the observer.^35^ In like manner, any clinical use of an ^192^Ir source that deviates from this calibration process should be considered. Specifically, the use of stainless steel interstitial needles may attenuate the source in comparison to conventional plastic catheters that are used to calibrate the source at the secondary standards lab and benchmark its source strength during a source exchange at the clinic.

While this commissioning work focuses on the use of the Elekta Prostate HDR brachytherapy system, there are other commercial products capable of similar image‐guided, intra‐operative HDR treatments that may follow a similar commissioning process. For example, Varian Vitesse (Palo Alto, CA) has been used for real‐time, US‐guided prostate HDR.^23^ While the recent literature has focused on LDR applications of the Vitesse (Varian Medical System, Inc.), both systems rely on an integrated framework to reconstruct needle position using US imaging. While a comparison of needle position accuracy between the Vitesse (Varian Medical System, Inc.) and the Oncentra Prostate (Elekta AB) is outside the scope of this work, it is clear that the commissioning of both systems would benefit from the integrated structure demonstrate.

### 4.2 Considerations of US artifacts and needle reconstruction

**FIGURE 17:**
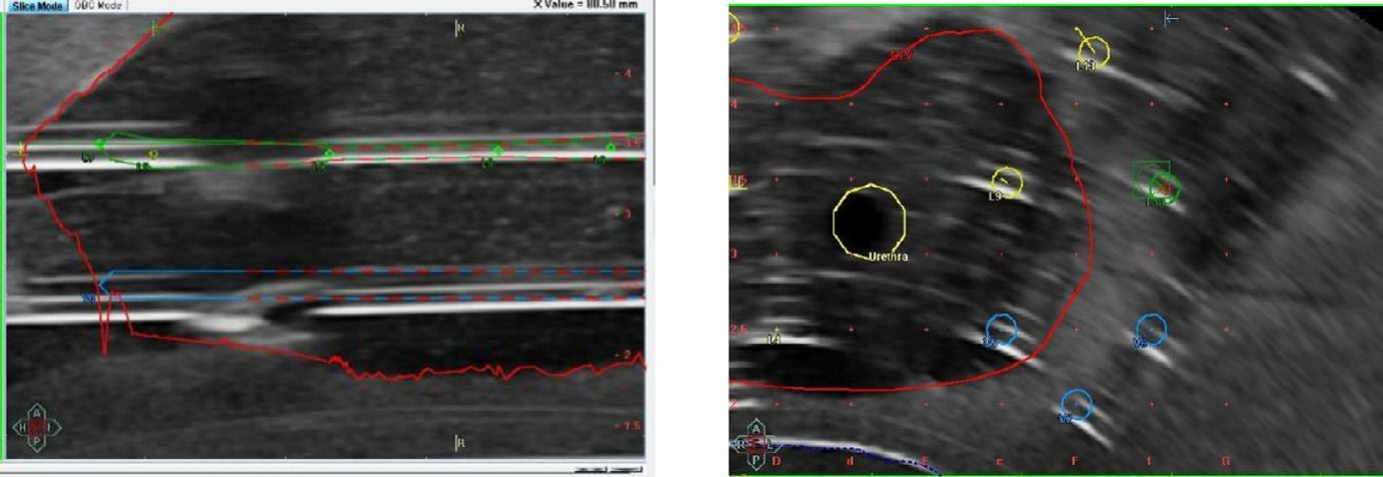
(Left) Sagittal view of an instance where a proximal needle induced signal loss via shadowing of two distal needles. (Right) Transverse view during needle reconstruction with several artifacts, including speed of sound and reverberation artifacts

The presence of imaging artifacts necessitates careful thought when reconstructing the needles as needle reconstruction appears to be one of the largest sources of uncertainty during the treatment planning process. In addition to these artifacts, needle reconstruction is further complicated by the fact these artifacts appear simultaneously. The images included in this report illustrate the three artifacts (speed of sound, reverberation, and shadowing) as they manifest during a needle reconstruction exercise. User intuition in addition to artifact recognition is necessary to accurately reconstruct the needles. Figure 17 demonstrates a clear speed of sound artifact as a portion of the needle is systematically displaced away from the probe due to the presence of a bubble in the rectal balloon. A notable amount of reverberation is also observed distally, which can cause the user to mistakenly place a needle more distally one or more reverberations, especially if there are a superposition from multiple, proximal needles. It is also helpful to also make use of both the transverse and sagittal views to assist in reconstructing the needle's trajectory. In some cases, portions of the needle may appear to disappear due to a shadow artifact of a more distal needle or appear “pulled down” systematically due to a speed of sound artifact from a closer proximal needle. In some instances, the shadow of a needle as observed on the transverse view can be used to locate the needle's position.

## 5 CONCLUSION

The commissioning of real‐time US‐guided, prostate HDR systems requires careful planning and testing of both the imaging and treatment planning components. While some aspects of the system are well differentiated, such as the US probe's image quality or the dose calculation accuracy within the TPS, accurate needle reconstruction necessitates the proper functionality and accuracy of the system holistically. An integrated commissioning procedure was compiled from multiple task groups and adapted to include the checks necessary to benchmark the image reconstruction accuracy based on published literature. These additional tasks included the imaging resolution and accuracy, origin coincidence between imaging crystals, mechanical and electronic calibrations, and the longitudinal positioning accuracy of the US‐TPS system.

## CONFLICTS OF INTEREST

The authors have nothing to disclose.

## AUTHOR CONTRIBUTIONS

Blake R Smith, PhD was the primary researcher performing the specific measurement tasks, testing the clinical workflow, developing secondary check tools, and assisting in the formal documentation of the procedural workflow. Sarah A. Strand, PhD was a contributing researcher who helped perform several of the image‐based commissioning measurements. David Dunkerley, PhD was the primary physicist that had commissioned the RadCalc software and help facilitate dose calculation checks among the dose calculation engines. Abigail E. Besemer, PhD, had several collaborative contributions to the workflow development and measurement techniques that have been utilized at the University of Nebraska Medical Center. Dr. Besemer also had several editing contributions to the manuscript. Ryan T. Flynn, PhD, Jennifer D. Kos, Joseph M. Caster MD, PhD, and Bonnie S. Wagner, RN, and Yusung Kim, PhD had substantial contributions in the workflow development and clinical implementation of the HDR prostate procedure. These individuals also were responsible for the organization of materials, logistics of equipment, and personnel. In addition to the workflow development, Joseph Castor, MD, PhD, and Yusung Kim, PhD, had important roles determining the dose fractionation and healthy tissue tolerances that are used to evaluate HDR prostate treatment plans in this work. Yusung Kim, PhD, was the senior Medical Physicist that organized and oversaw the commissioning of the entire HDR prostate brachytherapy program presented in this manuscript.

## Supporting information

Appendix 1

## Figures and Tables

**FIGURE 18:**
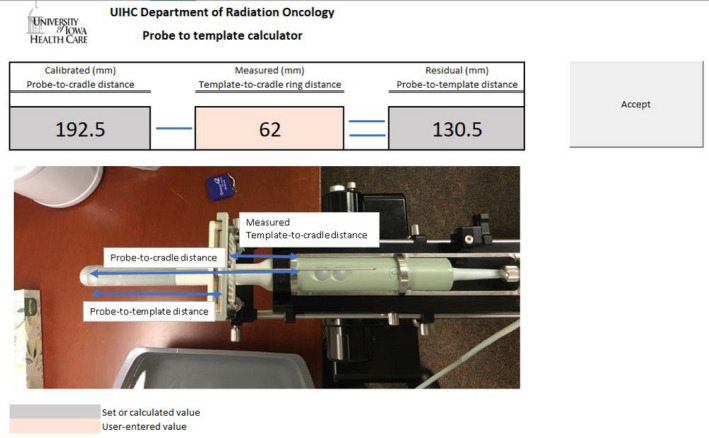
Probe‐to‐template calculator application to help facilitate intra‐operative, US‐guided HDR prostate brachytherapy treatments

## Data Availability

The specific workflow, checklists, and treatment analysis tools that were developed for the US‐guided intraoperative HDR prostate system are available as supplementary data and material to this article.

## 1 Probe‐to‐template calculator

Figure 18 shows the user interface from an application that was developed to facilitate probe‐to‐template distance calibration when the origin is set during a US‐guided HDR prostate brachytherapy procedure from the surface of the physical needle template. A picture is included that clearly illustrates the measurement distance and calculation parameters in a simple format that has been well‐suited for ease of use during the clinical procedures. Upon selecting “Accept”, the probe‐to‐template measurement is recorded in a historical recorded to help identify potential errors or measurement outliers.
