# Supplementary material for: Implementation of a real‐time, ultrasound‐guided prostate HDR brachytherapy program
Source: J Appl Clin Med Phys. 2021 Jul 26;22(9):189–214. doi: 10.1002/acm2.13363 (PMC8425918; doi:10.1002/acm2.13363)
Supplement: Supplementary file 1 — Appendix 1 [file ACM2-22-189-s001.docx]

## UIHC Prostate HDR Workflow with Instruction

## Overview

## This is a procedure workflow for performing real-time ultrasound (US)-guided prostate HDR treatments in the HDR suit using the Oncentra Prostate Treatment Planning System (OCP).

| **TERM** | **DEFINITION** |
| --- | --- |
| **RO** | Radiation Oncologist |
| **RN** | Registered Brachytherapy Nurse |
| **RTT** | Radiation Therapist |
| **P** | Physicist (Planner) for HDR Prostate Brachytherapy |
| **2P** | 2nd Physicist |
| **MPR** | Medical Physics Resident |
| **US** | Ultrasound |
| **OCP** | Oncentra Prostate |
| **LMB** | Left mouse button |
| **RMB** | Right mouse button |
| **MMB** | Middle mouse button |

## Preparation (Prior to the treatment day)

## This is a procedure workflow for performing real-time ultrasound (US)-guided prostate HDR treatments in the HDR suit using the Oncentra Prostate Treatment Planning System (OCP).

| **V** | **TASK** | **Staff** |
| --- | --- | --- |
|  | Schedule patient in the HDR suite (EPIC & MOSAIQ) and email secretary to block RO’s schedule | RN |
|  | RO gets patient consent during consult and RN files for upload | RO, RN |
|  | RN gives patient bowel prep instructions | RN |
|  | Ensure all equipment is available and cleaned or sterilized (See Equipment Checklist at Appendix G.) | MA |
|  | RTT checks the equipment/device that they need to set up on the night before prostate HDR treatment (See Equipment Checklist at Appendix G.) | RTT |
|  | RN and RTT ask Kelly to order any necessary supplies (e.g., sterile cover, stabilization needles, TRUS balloon, etc.) | RN, RTT |
|  | RTT locates the OCP cart and Stepper/Stabilizer at HDR Suite and locates the white attachment table at HDR planning room on the night before prostate HDR treatment | RTT |
|  | Connect all cables of the US, OCP Cart, and Stepper/Stabilizer and turn on US and OCP computer.   - Perform 3 QA for the connection and stepper functionality | MPR |

## Preparation, Room Set-up, QA (Day of treatment)

| **V** | **TASK** | **Staff** |
| --- | --- | --- |
|  | **HDR DailyQA must be done** at least 30min before its schedule | MPR |
|  | Set up US and attach TRUS probe  Attach black ring from stepper to TRUS probe prior to putting on the balloon   - Make sure right preset (Brachy Prostate L), probe (E14CL4b), and grid (User-Defined: BrachyHDR) | RTT |
|  | **Prep TRUS balloon**: Very IMPORTANT, now we use US images for treatment planning   - Fill balloon with ultrasound gel (swing to distribute) - Place a brachy balloon on the probe. - Ensure that all bubbles are pressed out - Retrieve a BD 60 mL Luer-Lock Syringe and fill syringe with 25-50 mL saline and partially fill the balloon holding probe tip down. - Fill and aspirate with 10-20 cc until all air is removed (VERY IMPORTANT). Press out any bubbles back towards the syringe. Pull back the plunger while the syringe is upright to pull bubbles out to the syringe. When one gets some air in the syringe, unattach, push out air, reattach. Repeat until most of the air is removed, which may require several attempts. - At the end, keep the 30 cc of saline in the balloon the goal is to consistently use 30 cc for all cases.   Clean up gel and tape down the balloon securely. Fold over the last part of the tape to allow it to be easily removed and make a courtesy tab for easy removal later.   - The balloon and TRUS probe can become accidentally separated through a series of in-and-out procedures. Then, the whole team needs to start from scratch by re-attaching a balloon, imaging, planning, etc. - Gel ready for the single lumen catheter | RTT |
|  | Prep micro-bubble contrast for single-lumen urethra catheters   - Two 50 mL syringes - 20 mL of KY jelly and 5 mL of air in one syringe - 25 mL of saline in the other syringe - 3 way stop cock - Connect to stop cock and push substances back and forth between the two syringes to create the bubble contrast | RTT |
|  | Double check US right preset (Brachy Prostate L), probe(E14CL4b), and grid (User-Defined: BrachyHDR) | P |
|  | Review total of 4 cable connections   - US cable to OCP - Two OCP cables to stepper - US and TRUS connection | P |
|  | Review all initial parameter set-up at OCP   - Validate treatment type, catheter sources, afterloader, source calibration | P |
|  | Check probe & depth detection at OCP   - Confirm US Probe is auto-detected as E14CL4b (S if in sagittal model and T if in transverse mode) and the depth as S6.0 (or T6.0) | P |
|  | **Check P drive** (Prostate HDR folder)   - Open DVH Eval excel @P   **Remotely connect to Planner’s office computer**  **Open MOSAIQ**   - DVH Eval excel can be found @S:\OncShare\PHYSICS\Elekta Prostate HDR - Getting ready MOSAIQ document: Plan note (draft), QCL, Needle configuration (doc), DVH Eval (excel) | P |
|  | **Check US and OCP grids align** on the screen:   - On the BK3000 US unit, hit the template button and then make sure the ‘User-Defined: BrachyHDR’ template. - In the OCP click the show/hide template button. - Confirm that the grey and red grids are aligned. If there is a rotational offset, rotate the probe a little bit within the slack of the mechanical lock.   **Rotate TRUS probe to match US and OCP grids**  **Set the mechanical lock**.  **Turn off template grid in the US but keep on the OCP**.   - Else it will burn into the 3D US image!   **Ensure a template support is fully proximal position**. | P |
|  | **Create new patient and/or study**   - The study names are; Boost Fx1 or Mono Fx1 (or Fx2) - Enter RO last name | P |
|  | **Ensure preference setting (HDR Boost or HDR Monotherapy) is correct** | P |
|  | Prepare patient prep items, sterile gowns, and other items | RN |
|  | Apply fleet enema before coming to treatment room | RN |
|  | **Set up a sterile table**.   - 1 pack of stabilization needles (2 needles/pack) - Sterile cover for patient - Sterile cover for a stepper - 1 prostate HDR tray and 1 backup prostate HDR tray - 1 prostate HDR tray includes;   - 20 stainless steel needles (240 mm)   - 20 sterilized obturators   - 1 diddler   - 1 obturator handle   - 1 metal ruler   - Parts of prostate HDR template | RN |

## Patient Preparation

| **V** | **TASK** | **Staff** |
| --- | --- | --- |
|  | **Bring in patient** and have them lie with their head at the top of the table. Do time out with anesthesia team, RTT and RO.   - Ensure the patients bottom is slightly over the inferior edge of the main Zephyr plate before he is asleep. Otherwise the RO might not be able to insert the probe deep enough and we may need to manually move the patient inferiorly. | RTT |
|  | **PPE Time Out with all staff in HDR Suite** | RN, RTT,RO,P |
|  | Attach arm board, stirrup and stepper holder to patient Left side of couch, aligned in the same way as Right side. | RN, RTT |
|  | Place patient in the dorsal lithotomy position, with legs in stirrups and pelvis aligned with the distal end of bed. Make sure the pelvis is symmetric and level. Remove the footboard.   - When the patient's legs are in stirrups, ensure the patient is located at the edge of Zephyr table. - Place chux under pelvis | RN, RTT |
|  | **Level the legs** in stirrups and if necessary, adjust each stirrup. Check that patient position is optimal. If legs need to be adjusted, RTT/RN adjusts. Position so that:   - The legs are symmetric about the rectum - Physics: The posterior aspect of the prostate is a maximum of 3 mm below row 2 of the US grid - The urethra runs straight along row G of US grid - If required, add more saline into balloon | RTT, P |
|  | **Set up TRUS with cradle, and ring to Stepper**   - Ensure to tightly assemble TRUS, cradle, and black ring | P |
|  | **Verify TRUS with cradle and ring to Stepper is properly constructed**   - **Validate pin of US probe is fully inserted in cradle**   **Validate ring is securely attached to stepper and can freely rotate** | P2 |
|  | **Set up TRUS with cradle, and ring to Stepper** | P |
|  | **RO generates a dummy Rx** per its sample as like GYN HDR but with no needle #s.   - This enables 2P to address the MOSAIQ related documentation | RO |
|  | **2^nd^ Physicist complete all MOSAIQ related task;**   - Brachy Plan note: draft - Faculty QA QCL - Create dummy field for virtual delivery - Schedule the treatment delivery in MOSAIQ calendar | 2P |
|  | **RO places single-lumen catheter and injects bubble solution** | RO |
|  | Secure up scrotum and penis with blue cloth to patient skin and 3M Loban 2. Prep patient from scrotum to anus with chlorhexadine 2% paint | RN, RTT,RO |
|  | Drape patient with U drape   - Use hemostats to clamp drape onto patient leggings if needed. | RO |
|  | Attach compression hose to SCD leggings | RTT,RN |

## Prepare US Position and Check Image Quality

| **V** | **TASK** | **Staff** |
| --- | --- | --- |
|  | **Assemble the template**:   - Test one needle and lock the template to confirm the template is assembled correctly and the locking mechanism is working. | RO |
|  | **Measure the height of the sterile template** from the bottom of the template leg to the bottom of the screw, and P will check.   - Both sides must be 23 mm (± 1 mm). - If the height is out of tolerance, the template is NO longer calibrated and needle insertion will NOT be as accurate, thus RO needs to open a backup Prostate HDR tray. | RO, P |
|  | **Drape stepper** with clear plastic (sterile) cover.   - Insert probe into the precut hole in the drape. - Put holes in the drape for the template holder | RO, RTT |
|  | **Insert template and tighten front screws on template holder**.   - Ensure all knobs on stepper are locked | RO |
|  | **Verify that template is correct orientation-grid metal to the MD** | P |
|  | **Add ultrasound gel to TRUS probe** | RTT |
|  | Insert TRUS probe into patient’s anatomy:   - Make sure height is unlocked - Advance the stepper along the floor stand superiorly until the TRUS probe is about to enter the rectum. - Lock stabilizer - Engage side lock of TRUS probe | RO, P |
|  | **Place US gel on US probe** | RTT |
|  | Advance the US probe into the rectum using the longitudinal black knob   - Its two small knobs should be unlocked on the stepper. | RO |
|  | **Final check that patient position is optimal**.   - The prostate is **symmetric** about the grid in the image on the US display - The posterior aspect of the prostate is a maximum of **3 mm below row 1** of the US grid - **The urethra runs straight along column D of the US grid** - RO can adjust lateral movement knob to make the prostate symmetric - If required, add more saline into balloon - If the patient’s legs need to be adjusted, a non-sterile person will need to reach under the drape to adjust. | RO, RTT, RN |
|  | Adjust the stepper/TRUS probe position so that there is good contact, and that one can visualize the prostate well:   - Extend the template holder out to ~1 cm from the perineum (leaving a little space for using the diddler if needed) - Ensure that the TRUS probe can be advanced at least ~ 2 cm that can be checked on OCP - Note: Greater than 2 cm is ideal since it is possible that the prostate will be pushed superiorly during the procedure, and reconstruction of all metal needle tips requires 9 mm of over-travel distal to the prostate. - Ensure that the prostate is centered in the template in the R/L direction. This can be checked by ensuring the urethra is aligned with column D of the US grid - Inflate the TRUS balloon or adjust the stabilizer height to position the prostate in the A/P direction. Ideally, the second or first row of the template falls a little above the posterior border of the prostate. This enables the addition of dwell positions while minimizing rectum dose. - Ensure there is good contact with the probe from the apex past the base by checking sagittal images and when rotating the probe ± 90 degrees through the full rotation. If not, adjust the probe positions, TRUS balloon filling again, check the brachy balloon for bubbles. - Adjust US settings if needed to improve image quality. - Check for pubic arch interference. If there is pubic arc interference, consider adjusting patient’s leg positions now. Otherwise there may be needle deflection or some anterior needle positions that are unusable. | RO |
|  | **Note**:   - If at any point later in the procedures the legs are moved or the balloon is adjusted, be sure to reset the base (origin) plane and re-image so that the needle depth is accurate. - **Whenever a ‘Base plane’ is moved, one must reset the origin. Whenever the origin is reset, then one must re-acquire the planning images.** - If there is gas in the rectum or a bubble in the brachy balloon pops after the template is in place, you can remove the probe by sliding off the stepper platform to have better access to the rectum and to adjust/redo the brachy balloon. | P |

## Pre-Needle-Insertion Virtual Treatment Plan

## 4-1. Initial Image Acquisition

| **V** | **TASK** | **Staff** |
| --- | --- | --- |
|  | **Set Origin (Appendix B.)**:   - Go to **Live Plan and Live Imaging** (US probe icon) - Go to **transverse mode** of US and move probe to the base plane - RO adjust the physical **longitudinal depth indicator knob to ‘0 (zero)’**. This will be a sanity check in case the base plane moves. - RO check the mechanical lock (black) - Ensure that **the grids align** between OCP and US - **Uncheck the grid** at OCP and US - **Verify the US probe (E14CL4bT) and depth (T6)** - **Click Keep motor at position** - **Click ‘Motorized acquisition’ and ‘Motorized navigation’** - **Click 'Set Origin’**   - Click ‘OK’ to message about lock and being set successfully - RO measure the D= “distance from the white base plate to the proximal edge of the most distal silver ring on the cradle“ in mm. Do not move the probe until the value is entered.   - P validates the measured distance while RO measures - **RO release the mechanical lock (black)** - **Adjust image focus and optimize gain to have better contrast**   - In Sagittal scan, should be performed in sagittal US view with prostate centered     - In Transvers scan, then in transverse US view   - Adjust gain to provide good prostate contrast with surrounding tissue   - If necessary, adjust field of depth to anterior portion of prostate | P, RO, RTT |
|  | **Acquire Radial Scan (Sagittal)**:   - RO go to **sagittal mode** (click the proximal button) on TRUS probe - **Advance TRUS** so that there is a little more space - Ensure that the TRUS probe can be advanced at least ~ 2 cm past the prostate. - **If for some reason you can’t get the probe deep enough** to image distal to the base plane and needles are cut off, unfortunately you can’t activate dwells or calculate dose outside of the image. The dose grid won’t expand outside the image.   - Take TRUS probe out and make sure - **Click the Start Scan button**. Click acquire 3D-US image button | P, RO |
|  | **Acquire Translational Scan (Transverse)**: When the prostate is long enough or ECRM motor does not work   - Make sure you’re in Transverse mode on the US display - Advance the probe so that the transverse plane is 10-20 mm past the base plane (about 3-4 clicks of the longitudinal knob). - Ensure that the stepper is in continuous and not stepping mode: unlock big silver sub-knob of longitudinal black knob - Click the Start Scan button. Click ‘Acquire 3D-US Image’ button - Have the RO very slowly pull the probe inferiorly to manually scan (Color bar represents its speed that should be green) - Once finished, click the Stop Acquisition. | P, RO |
|  | If image needs to be rescanned click delete acquisition and repeat steps | P |

## 4-2. Initial Contouring for the Virtual Plan

| **V** | **TASK** | **Staff** |
| --- | --- | --- |
|  | Select **VOI Handling** workspace | P |
|  | **Scroll through the image** ensuring that all relevant components are contained in the image and that the image quality is acceptable.   - If needed, adjust the window/level of the image | P |
|  | **Set the apex and reference plane** (by clicking ‘Plane Definition’)   - Make sure to unclick “Plane Definition” when you are done.   Do NOT adjust the base plane since the origin was set prior to imaging.   - If the base plane is off, reset the origin and re-acquire the image. If you don’t re-image after resetting the origin, the reset origin won’t be valid. | P |
|  | It will ask you to save the changed data to the data base. Click YES | P |
|  | **Contour draft Prostate (= CTV)** for Virtual Plan   - RO contours - Contour on base, apex, reference, two slices in between and if necessary, couple of more slices (~ 7-8 slices)   Create PTV: if RO wants to add 2 mm margin to CTV (= prostate)   - Go to margin tab, select CTV, and make sure the default margins are;   - -X (Patient Right): 2.00 mm and +X: 2.00 mm   - -Y (Anterior): 2.00 mm and +Y: 2.00 mm   - -Z (Inferior): 1.00 mm and Z: 0.00 mm - Click Apply and Yes to message and wait - Click the created Margin contour and select options. - Rename to PTV in window and select ‘CTV’ (its VOI type will be CTV2). - In VOI attributes, Multiple type will be checked (Do not click ‘Active target’) - Click OK | RO |
|  | **Contour the urethra** using circle   - Contour on base, apex, reference and if necessary, add 1-2 slices in between. | P |
|  | **Contour the rectum** using pearl   - Prioritize anterior portion of the rectal wall | P |
|  | Note: If an erroneous contour occurs, it is often best to clear the contour (by clicking ‘Clear Contour’) on the current slice and restart   - If a contour exists above and below, an interpolated contour will be inserted by clearing the current layer’s contour | P |
|  | **For a subsequent treatment fraction, click ‘File operations’ (floppy disc) tab**  **Load previous structures** from other study onto recent scanned image  Ensure that the desired patient and study is selected before proceeding (which may be different than the study ID that you copied your contours from)   - All changes from this point on will be saved to the patient and study ID that is selected   Modify VOIs by selecting, “Modify VOI” button   - If CTV is adjusted, re-generate PTV structure (if RO wants PTV) | P |

## 4-3. Normalization and Prescription

| **V** | **TASK** | **Staff** |
| --- | --- | --- |
|  | Go to the **Normalization and Prescription** workspace | P |
|  | Click Yes (when a message will prompt you to save). Type ‘Initial structures’ for the comment window  Click OK | P |
|  | **Confirm that the prescription** is correct per MOSAIQ   - HDR Monotherapy: default to 13 Gy - HDR Boost: default to 15 Gy | P |
|  | **Click “Set current button”**   - This is to update the activity and date to the current values. Confirm that the current date and time are correct. | P |

## 4-4. Create Virtual Pre-Plan

| **V** | **TASK** | **Staff** |
| --- | --- | --- |
|  | Go to the **“Catheter Placement” workspace** | P |
|  | **Enable needle grid overlay**  **Enable catheter visualization and dwell activation visualization**  **Go to ‘Reference plane’**  **Click ‘Insertion’** | P |
|  | Place needles using the standard loading pattern (Appendix A.) as a starting point   - Can add more needles, especially anteriorly to help account for swelling and needle placement uncertainties. It is easier to add more needles now and not use them than have to add more later. - Do peripheral loading, considering prostate swelling~~.~~ - If necessary, delete the initial needle positions by selecting ‘Delete’ - **Use 14 to 20 needles** - Needle increasing number should be from left to right and down to up.   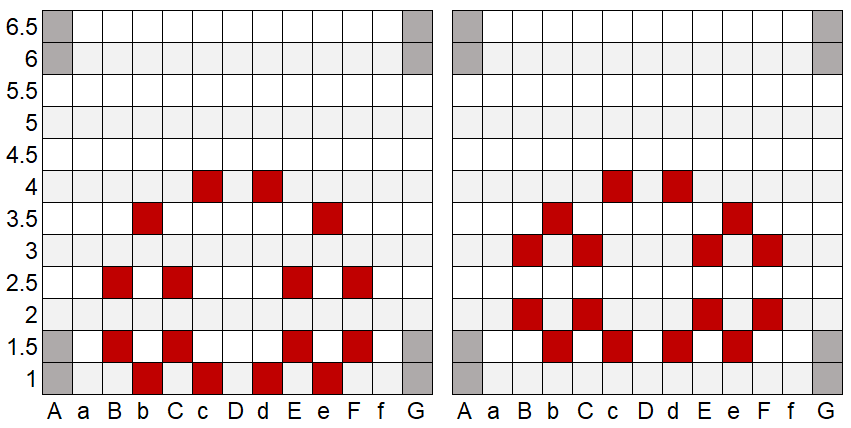 | P, RO |
|  | Go to the **“Dose Optimization”** tab | P |
|  | Select the “Inverse Optimization” Tab and ensure that the DVHO is selected. | P |
|  | **Click the “Optimize” button**  Click calculate and update tabs on bottom right.  Calculate and update isodose lines for visualization.  Click the icon of ‘View isodose’ | P |
|  | **Evaluate the dose monitoring metrics and isodose lines**. The following are the dose constraints for the dose monitoring lights:   - CTV: V100% > 95% - CTV: V150% < 35% - CTV: 100% ≤ D90 ≤ 115% - Urethra: V115% < 5% - Rectum: D2cc < 70%   If the plan is not meeting the objectives:   - Adjust the optimization objectives first - Try to do graphical optimization or manual optimization - Change needle location or add more needles | P, RO |
|  | **Save**   - Once satisfied with the plan, confirm with the RO that the dose distribution is acceptable for the pre-plan. - Go to ‘File operations’ - Click ‘Save Plan to SB (SmoothBase)’ - Type ‘Initial plan’ when a message pops up | P, RO |
|  | **Choose two unused grid positions** that can be used for the stabilization needles and tell the RO | P |

## Needle Insertion/Live Needle Reconstruction

## 5.1 Initial Push

| **V** | **TASK** | **Staff** |
| --- | --- | --- |
|  | **Inform anesthesiologist that needles will soon be inserted** | RO, P |
|  | Adjust overhead lights to Physician’s preference and optimal visualization | RTT |
|  | Switch back to the **Live Plan and Live Imaging**  Go to **Sagittal imaging mode**.   - The 3D axial image should be on the right with the red radial position line - To set the left image to live **click “Live Image”** on the right sidebar (where the start scan button usually is). - **Unload previous image** to view real-time images | P |
|  | **Unclick isodose line view and isodose update** (to free computer memory)  **Unclick dose update in the monitoring signal panel** (to free computer memory) | P |
|  | RO **unlocks the mechanical lock**. | P, RO |
|  | **RO inserts the two stabilization needles** at the agreed-upon positions. Use the red radial line on the 3D image to rotate to the grid position for the stabilization needles. | RO |
|  | **Check that “keep motor at position”** is checked.   - Later, uncheck “keep motor at position” whenever a physician needs to rotate the probe. | P, RO |
|  | In the Transverse 3D view (left panel), **click on the first needle** and the motor will rotate the US to point at that needle.   - If the radial line is bouncing that means the ‘Keep Motor at Position’ button is not checked. - RO can advance the TRUS probe to locate the distal needle position. - **Adjust virtual needle position before CALL OUT**   - so that the first dwell position (red) properly covers the base plane. | P |
|  | **Call out the needle template location** and needle insertion:   - Use the NATO alphabet **Alpha, Bravo, Charlie, Delta, Echo, Foxtrot, Golf** (“Big Charlie 4.5” (C 4.5) or “little Delta 5” (d 5). - Needle implant order should be left to right, top to bottom - RO inserts needle while watching it advance in the sagittal view - When RO looks up to screen read out the virtual needle number (e.g. V16) so they know where to look in the Sagittal view - If the needle doesn’t go where expected, the RO may use the diddler to adjust. | P, RO |
|  | Live Reconstruction:   - **DO NOT adjust virtual needle position until RO has inserted the needle all the way** after initial adjustment - **Click on the catheter and set it to live (green)**, once it’s fully inserted. - Click on the tip and drag it to roughly match the actual needle position.   - Don’t spend too much time reconstructing needles now because the prostate may move as more are inserted and when the obturators are removed.   - Focus on the longitudinal reconstruction. - When finished **click on the next needle** and the first needles should turn to yellow.   - Click ‘ctrl and click a spot in a green needle’ to manual complete the live reconstruction. | P |

## 5.2 Final Push and Obturator Removal

| **V** | **TASK** | **Staff** |
| --- | --- | --- |
|  | **Go through each of the needles** and ensure the tip is at least ~11 mm past the base plane (if possible) to ensure good superior coverage | P |
|  | **Partially (halfway) lock** the template with fixation screwdriver | RO |
|  | **RO will remove the obturators** one-by-one while the physicist adjusts the live reconstruction. If the needles are difficult to see, have the RO wiggle the obturator. Fine tuning the needle reconstruction now will make it easier to identify needles in the 3D image since the needle movement after the obturators are removed will be taken into account. | P, RO |
|  | **Remove the stabilization needles** if necessary   - They will create artifacts and may be confused with needles in the 3D image but if there is great concern about prostate movement, they can be left in for the 3D imaging | RO |
|  | **Fully lock** template and set the mechanical lock | RO |
|  | **Verify Needle Template is locked!** | P, 2P |
|  | **Uncheck ‘Keep motor at position’**  **Rotate the TRUS probe to a central slice** in sagittal mode and check to see if the prostate has shifted superiorly.:   - If there is a large deviation, RO go to **transverse imaging mode and go to the base (origin) plane** - Check if the longitudinal depth on the physical depth indicator on the stepper is still ‘0(zero)’ | P |
|  | **Inform anestheiolopgist that needle insertion is complete** | RO, P |

## Post-Insertion Treatment Plan

## 6.1 Final Image Acquisition

| **V** | **TASK** | **Staff** |
| --- | --- | --- |
|  | **Before imaging,** ensure all knobs on stepper except for two small knobs on the longitudinal movement knob (black) and template are locked. | RO |
|  | **Final check that prostate is still good to scan**.   - The prostate is **symmetric** about the US grid - The posterior aspect of the prostate is a maximum of **3 mm below row 1** of the US grid - **The urethra runs reasonably straight** along row G of US grid - RO can adjust lateral movement knob to make the prostate symmetric - If required, add more saline into the balloon - If the patient’s legs need to be adjusted, a non-sterile person will need to reach under the drape to adjust. | RO, RTT, RN |
|  | **Set Origin (Appendix B.)**:   - Go to **transverse mode and move probe to the base plane**   - **Check the physical longitudinal depth indicator knob**.   - If it has moved from the original 0 setting than the base plane has moved.   - RO adjust the physical **longitudinal depth indicator knob to a ‘0 (zero)’**. - RO check the mechanical lock (black) - Ensure that **the grids align** between OCP and US - **Uncheck the grid at OCP and US** - **Verify the US probe (E14CL4bT) and depth (T6)** - **Click Keep motor at position** - Make sure to **click ‘Motorized acquisition’ and ‘Motorized navigation’** - **Click set Origin**   - A message appears reminding you to set the mechanical lock-> ok. - In the ‘Base plane changed’ window that appears;   - **Check box for contours**   - **Uncheck boxes for virtual & live catheters** (already reconstructed to the correct depth so you don’t want to adjust them.).   - Click OK - RO measure the D= “distance from the white base plate to the proximal edge of the most distal silver ring on the cradle“ in mm. Do not move the probe until the value is entered.   - P **validates the distance while RO measures** - **Have the RO release the mechanical lock (black)** - **Check and adjust US gain and field of view** | P, RO |
|  | **Acquire Radial Scan (Sagittal)**:   - RO go to **sagittal mode** (click the proximal button) on TRUS probe and adjust the FOV so that there is a little more space ~~(at least more than 15 mm)~~ near the base than the apex. - **Double check that the mechanical lock is released**. - **RO manually rotate TRUS probe** to ensure all needle tips are included in sagittal US images with 2-3 mm margin. - **Check marks for**;   - Motorized acquisition   - Motorized navigation   - Keep motor position - **Click Start Scan**. - Click Acquire 3D-US image button | P, RO |
|  | **Acquire Translational Scan (Transverse)**: When the prostate is long enough or ECRM motor does not work   - While still in the **Transverse mode**, advance the probe so that the transverse plan is 20 - 30 mm past the base plane. The number on the longitudinal movement knob should be lower than 80. - Ensure that **the stepper is in continuous** and not stepping mode (unlock big (silver) knob of longitudinal black knob. - Click **Start Scan button**. Click acquire 3D-US image button - **RO very slowly pull the probe inferiorly to manually scan** (Color bar represents its speed that should be green) - Once finished, **click the Stop Scan**. | P, RO |

##

## 6.2 Final Contouring

| **V** | **TASK** | **Staff** |
| --- | --- | --- |
|  | Select **VOI Handling workspace** | P |
|  | Click yes in response to the message of “Unload the previous study?” This is the previous plan and image).  Click Yes for the question of saving   - Type **‘Post-insertion Image’** when asking for a comment - Click OK   Click Yes when the “Load VOIs” window will then appear asking if you want to load the previous VOIs.   - **Do NOT check the “Keep currently loaded VOIs” box** since that will cause duplicate ROIs that you may not be able to delete and will mess with the optimization setting. - The “Plan Loading” window will appear with the complete plan selected.   Click OK. | P |
|  | **Scroll through** the image to ensure that all relevant components are contained in the image and that the image quality is acceptable.   - If needed, adjust the window/level in the image | P |
|  | **Set the apex and reference plane** (by clicking ‘Plane Definition’)   - **Do NOT adjust the base plane**-the origin was set during prior to imaging. If the base plane is off, re-set the origin and re-acquire the image. (If you don’t re-image after resetting the origin, the reset origin won’t be valid.) | P |
|  | **Generate Needle Configuration (excel)**   - Print it | P |
|  | Adjust contours;   - **Physics click interpolated contour for CTV on each slice** - **RO review and adjust CTV, urethra, and rectum** - If RO wants, a couple of the most proximal slices of the bladder - Create PTV: if RO wants to use margin - Go to margin tab, select CTV, and make sure the default margins are;   - -X (Patient Right): 2.00 and +X: 2.00   - -Y (Anterior): 2.00 and +Y: 2.00   - -Z (Inferior): 1.00 and Z: 0.00 - click Apply and Yes to message and wait - Click the created Margin contour and select options. - Rename to PTV in window and select ‘CTV’ (its VOI type will be CTV2). - In VOI attributes, Multiple type will be checked (Do not click ‘Active target’) - Click OK | RO, P |
|  | **Measure free lengths using the needle configuration sheet (Appendix C.)**:   - RTT read off the first needle position and 2P measures that needle. - RTT records the length in mm on the needle composition sheet. | 2P, RTT |
|  | Once finished final contouring, **scroll through** all slices and check the contours.   - You can also look in the 3D mode in the upper right window. | P |
|  | **Double check free lengths by 2P and RTT**;   - 2P double check free lengths for the needles identified by Planner (Physics) if they are more than 2 mm off through needle reconstruction. - RTT record the new free numbers on the sheet | 2P, RTT |

## 6.3 Prescription

| **V** | **TASK** | **Staff** |
| --- | --- | --- |
|  | Go to the **Normalization and Prescription workspace** | P |
|  | A message will prompt you to save. Click Yes. For the comment window, enter **“post-insertion image”** and click OK. | P |
|  | **Confirm that the prescription** is still correct per MOSAIQ Rx;   - HDR Monotherapy: default to 13.5 Gy in two fraction - HDR Boost: default to 15 Gy | P |

## 6.4 Catheter reconstruction

| **V** | **TASK** | **Staff** |
| --- | --- | --- |
|  | Go to the **“Catheter Placement” workspace** | P |
|  | **Uncheck the update at the bottom right to stop auto-calculations** | P |
|  | **Turn off the display of the contours, active, and inactive sources**.   - To help better see the needles | P |
|  | **RMB click on the axial view and click go to APEX plane**;   - Since the apex is the closest to the template, the needle position is likely to be closest to the template grid position so the needles should be easier to identify. - Before, reconstruction, view the whole image and see if you can identify roughly where all the needles are. There may be some confusion if you just go one by one and don't look ahead where the other nearby needles are. - If the needle depths are too shallow, you can adjust the free length in the OCP TPS. Then if that helps, RO push in the needle more with half-locking the template and using the ruler, RO measure the desired free length (Appendix C.). - Start with the first needle by number (e.g. L1) and work your way anteriorly. (The posterior needles will be easier to see so you want to be sure of those first before reconstructing the most anterior ones--which are harder to see.) - Use RMB to move between screens | P, (RO) |
|  | On the right toolbar, click the **“Manual” button under reconstruction**   - Select Settings: Ensure Source Step is 2 mm | P |
|  | **Reconstruct all needles**;   - Scroll through transverse images from apex plane and ensure the reconstructed needle (green circle) match the brightest needle image - Add a node (green dot) to reconstruct the needle on axial US image if the trajectory is off. - On sagittal images, tune the needle tip by dragging the tip to the brightest needle image.   - RMB click to change to the sagittal view (RMB avoids dropping a point)   - LMB and hold the reconstruction point while using your middle finger to scroll. Then let go when you are on the desired slice. - Generally, 2-5 points are enough to reconstruct the metal needles if it’s straight. However, if there is a bend in the middle you may need to add additional points. - Repeat for all needles | P |
|  | **Review reconstruction**:   - Briefly go through them again and review the reconstruction - Zoom in on the 3D Mode and turn off the visualization of everything but the catheters check that the needles appear relatively straight and there are no odd kinks - Check that any needles that may appear notably shorter or longer than the others (shown in the bottom window) and ensure that is a real phenomenon and not just an error in the reconstruction - Once all catheters are reconstructed, enter Free Lengths into table at bottom of OCP screen. Once a value is entered, it will automatically lock the catheter. | P |
|  | **Enter measured free lengths into free length column** in the bottom table for each needle:   - Type in the measured distance - Ensure that the change is reasonable within 2 mm and that visually the needle tip position did not change drastically in the sagittal view.   **When free length is quite off**, see if there is a reason such as the needle extending beyond the image or the signal dropped out near the tip.   - **If the difference is larger than 2 mm, RO remeasure the distance again**. | P |
|  | **When reconstruction completed**   - 2nd Physicist and RTT ensure   - **Template is locked**   - **All the knobs on the stepper are locked** - 2^nd^ Physicist and RTT check all channel lengths   - Needle + tranfer tubes = index length **1229 mm**   - **Record them** on the prostate needle configuration sheet. - **2^nd^ Physicist connects all the catheters to the Flexitron with the RTT**   - When all the needle reconstruction and their free lengths are finalized   - When P completes needle reconstruction, P can let 2P start 2^nd^ physics check, while P covers this. | 2P, RTT |
|  | **RO perform final check**   - On the connections of catheters to Flexitron with RTT | RO, RTT |

## 6.5 Dose Optimization and Evaluation

| **V** | **TASK** | **Staff** |
| --- | --- | --- |
|  | Go to the **“Dose Optimization”** tab | P |
|  | **Add reference points** for RadCalc dose calc (BEFORE OPTIMIZATION):   - Click “Show Special Point List”-> “Add point” - Add 6 points in relatively homogeneous regions at base, reference, and apex slices but at least 1 within the urethra - When finished, click ‘Close’ - Note: If you don’t add these points before optimizing, there is a bug that they will not save as a part of the DICOM file and the second check calculation will not work. If you forget, add them, optimize (without changing anything), re-save and reapprove the plan, export the plan to the RadCalc again. | P |
|  | **Optimize plan**:   - Select the “Inverse Optimization” and ensure that the DVHO is selected, and click the “Optimize” button | P |
|  | **Turn on Activate/Deactivate Dose Update and click on Update box** on bottom Right screen located by the traffic lights   - Ensure DVH mode is not selected (located in Upper Right window of screen). This will help to speed up optimization. - Select Dose Settings/Isodose Visualization - Select calculation tab - Select dose grid resolution HIGH and click calculate | P |
|  | Go to the **“Dose Evaluation” tab** and calculate dose:   - DVH calculation will start automatically - Click Update button to calculate the isodose lines - Check the update box next to the dose monitoring metrics   **Evaluate the plan using DVH Eval excel**;   - Open “UIHC_Patient_Dose_Tracking” excel sheet @P:\Elekta PROSTATE HDR\ - Make sure yellow cells that manually typed are filled - Copy the dose stats form the DVH tab in OCP into the “Raw Oncentra Brachy Output” sheet of the excel   If all DVH parameters are good, review an isodose distribution. | P |
|  | **Note**: If the plan is not meeting the objectives:   - Adjust the optimization objectives - If you cannot get a good plan with the inverse optimizer, you can use graphical or manual optimization, but this should be a last resort.   - Graphical Tab-> Slider bar to local -> Optimize -> MMB to drag the isodose lines   - Manual Tab-> Dwell times (for the table) or Bar graph (for bar graph editing) - If needed you can expand the dwell position margin around the CTV   - Go to activation tab-> Settings   - Change the margin around CTV (default is 5 mm) to something large enough to include the catheter you want to activate   - Click Apply. You should see more dwell activated.   - Re-optimize--now it should use those dwell positions   If nothing works, add more needles or change needle (ONLY IF REALLY NECESSARY) --must unlock the template and reimage | P, (RO) |
|  | Once satisfied with the plan, **RO final review of DVH parameters and isodose lines** of the plan | RO, P |
|  | **Save the plan and add comment of “MD approved”**   - Go to the File operations (floppy disk icon) - **RMB on the yellow plan on Upper Right window. Select Approve (LMB))** | P |
|  | **RO update Rx note** and **‘MD Brahcy Plan Approval’ on MOSAIQ** | RO |
|  | **Print plan PDF**, select “Treatment Plan” from drop down menu | P |
|  | **Export the plan to the RadCalc** | P |
|  | **Run RadCalc second dose calculation check**   - Log-on to MNC309 in HDR treatment planning room - Run RadCalc.exe in RadCalc\\V: drive (map drive if not currently available) - RMB “import from RTP” and RMB plan from list - RMB “import selected” and double RMP on plan calculation - Verify Calculation parameters:   - Source model and calibration - RMB “compute point doses” - Create report   - RMB “print”   - Select “summary layout”   - Select “print to file” and “png” from drop-down list   - Verify path is Y:\RadCalc Export   - RMB “print” | 2P |
|  | **MOSAIQ Document: Check and do if not done**   - QCL - Schedule its treatment - MU Verify - BrachyPlan: including DVH evaluation - BrachyQA: including needle configuration - Other items described in 2nd physics checklist (Appendix F.) | 2P |
|  | **Export the plan to the Flexitron**   - Note, if the DICOM is not sending to the TCC, copy the DICOM file exported to the USB drive (available at OCP cart). Then at the TCC manually import the DICOM file. | P |
|  | **2^nd^ Physicist starts 2^nd^ check** | 2P |
|  | **Open patient on the TCC**, actualize dwells, and **print a pre-treatment printout** for second checker.  **Upload pre-treatment printout** in MOSAIQ  Review all MOSAIQ-related tasks   - Rx note update by RO - QCL - Virtual treatment schedule - Update Brachy plan note   **Run ‘Check-cables’** to address any obstruction issue. | P |
|  | Physicist and 2^nd^ physicist compare the Flexitron printout to Oncentra Prostate (TPS) printout | P, 2P |
|  | Perform or check radiation safety related tasks;   - Survey the Flexitron and the patient - Open Flexitron door to access emergency wheel - Position the pig, tweezers, and wire cutters near the patient and Flexitron - Make sure ‘no-go-through’ signs on each door. | P, RTT |
|  | Ensure all monitors and equipment is ready for anesthesia to monitor patient outside the HDR suit | RTT |
|  | After physics second check, **P and RO sign off** on the treatment delivery printout from TCC on MOSAIQ. | P, RO |
|  | Time out with P, RTT and RO before delivering tx | RO, RTT, P |
|  | After delivery, **print out post-treatment report** from TCC and P and RO sign off on the document in MOSAIQ. | P, RO |

## POST-Treatment

| **V** | **TASK** | **Staff** |
| --- | --- | --- |
|  | Physics will survey the patient, afterloader, room | P |
|  | Disconnect all transfer tubes and remove needles from the patient | RO |
|  | Remove the template from the stepper | RTT |
|  | Put the yellow caps on the needles  Run tap water on the template and needles.  Count and make sure there are;   - 20 used and unused needles. - 20 used and unused obturators.   Place them in a dirty biomed bin, soak instruments in Hydrogen Peroxide per instructions  Take the dirty biomed bin to the Rad Onc Soiled utility room   - See the sterilization and cleaning instruction in Appendix H. | RN |
|  | Apply pressure to the perineum to stop bleeding | RO |
|  | Remove drapes from patient and bring legs down | RTT, RN |
|  | - Remove the drape from the stepper - Remove the template support arm, a silver cradle, and a black probe holding ring from the stepper head. - Remove the template locking screws (two of them) from the support arm. - Remove the BK adapter plate and put them into a dirty biomed bin. - Disassemble the silver cradle from the motor / ECRM and the black probe holder ring from the cradle. - Clean them with the non-alcohol intercept wipes - Use the brushes to thoroughly clean the template locking screw holes (this is where blood can easily get stuck). - Wipe down and clean a silver cradle and a black probe holder ring using the non-alcohol intercept wipes. - Be sure to get all of the blood off from the rest of stepper head using the non-alcohol intercept wipes. - May also need to wipe down with the black wipes or a paper towel afterward to remove any white residue that the bleach wipes might leave. - See the sterilization and cleaning instruction in Appendix H. | RTT |
|  | - Thoroughly wipe down the motor and ECRM carefully using the black wipes. - Use either the non-alcohol intercept wipes and wipe outside of the encoder, the whole cable and connector, and magnetic strip. - Do not place the encoder connector in any solvent or liquid (wipe off only). | RTT |
|  | - Should wipe down the US cable (DVI) using Non-alcohol Intercept Wipes (blue and white container) connecting to the Oncentra Prostate cart, along with cleaning the BK3000 US unit - Disassemble the TRUS probe from the stepper head and put it in a disposable tray. - Clean the TRUS probe with the Sani Cloth Plus Germicidal Wipe (Red Top) - Clean the US cart - Take the disposable tray for the TRUS probe in the Rad Onc Soiled room. | RTT |
|  | Wipe down transfer cables with alcohol wipes  Disconnect cables from the stepper, ECRM motor, and BK3000 US unit   - Wipe down the cables attached to OCP cart - Move the OCP cart in the HDR planning room | P |
|  | Perform all items in RTT Checklist (Appendix D.) | RTT |
|  | Perform all items in Physics Planning Checklist (Appendix E.) | P |
|  | Bill:   - “Bill HDR Treatment” task - 1 x C1717 (HDR Source Charge) - 1 x 55875 (Trans-perineal placement of intestinal needles) - 1 x 76965 (Ultrasound guidance for interstitial radioembolization) - 1 x 73318 (Brachy iso complex >12 catheters) - 1 x 77772 (HDR Brachytherapy >12 catheters) - # x C1715 (# of stainless-steel needles + # of stabilization needles) | Jana |

## APPENDICES

Appendix A – Standardized Needle Placement Diagram

Appendix B – Basic 3D US Scan Steps and Basic Operation of Stepper and Stabilizer

Appendix C – Free Length Calculation in Oncentra Prostate (OCP)

Appendix D – RTT Checklist

Appendix E – Physics Planning Checklist

Appendix F – 2nd Physics Checklist

Appendix G – Equipment Checklist

Appendix H - Sterilization and Cleaning Instruction

**Appendix A. Standardized Needle Placement Diagram**

**
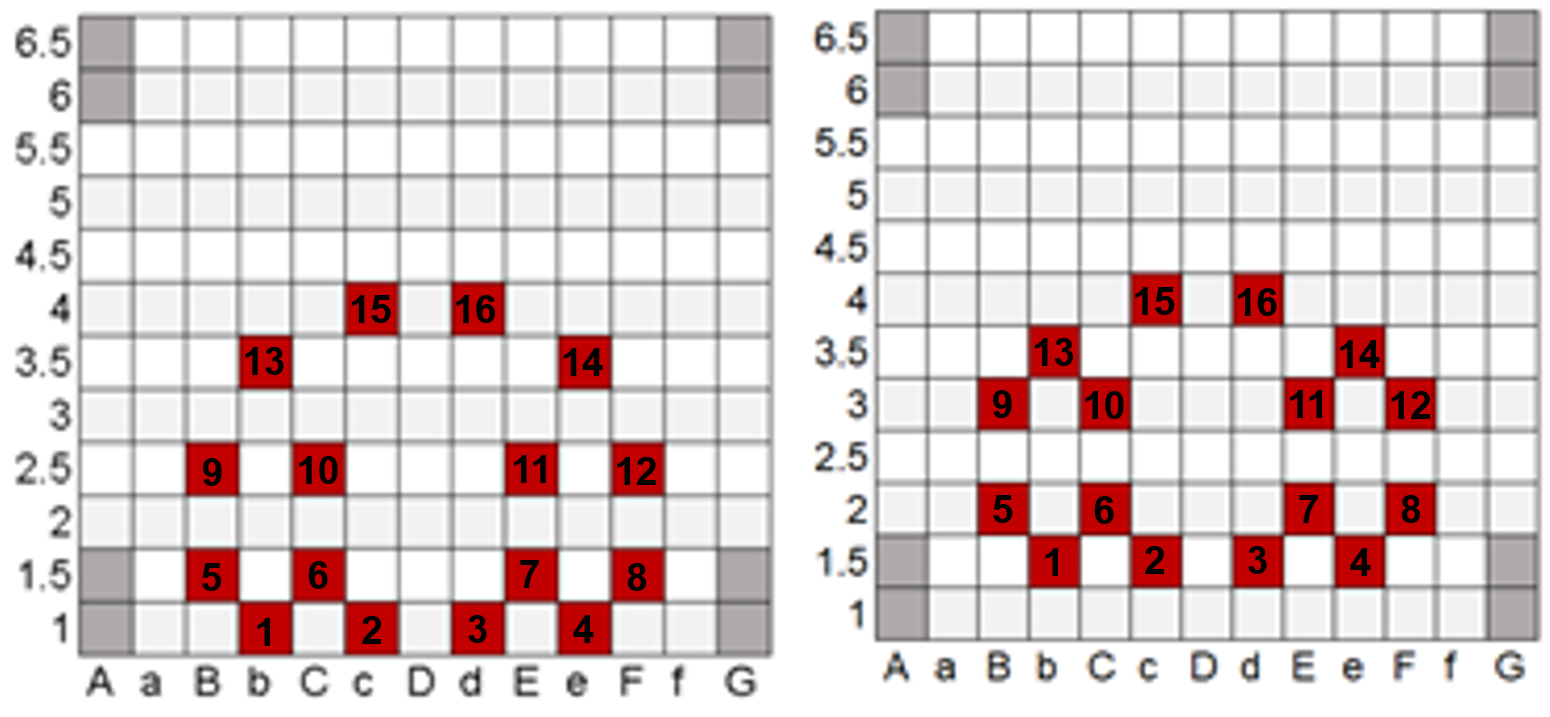
 A**lpha **B**ravo **C**harlie **D**elta **E**cho **F**oxtrot **G**olf

Needles inserted using a standardized template for positioning and to a depth based on anatomy. See diagrams for standard needle placement. Each red box represents needle position. The number in each red box represents each channel number of Flexitron where its transfer guide tube is connected. For an instance, RTT and second physicist connect channel # 1 tube to the #1 needle in the diagram. However, the order of needle insertion is opposite. RO inserts the first needle from left to right and top to bottom. For an example, the # 15 needle in the diagrams will be the first needle that RO inserts.

An arrangement of 16 catheters is suitable for most patients. Catheters are placed symmetrically. The anterior 4 catheters define the anterior anatomic border of the prostate at the mid-plane. The next 2 rows of catheters are placed approximately 1 cm apart, with the medial catheters 1 cm away from the urethra. Ideally, the fourth and final row is 5 mm away from the posterior border of the prostate. This arrangement of catheters allows for consistently high target coverage with adequate sparing of the urethra and rectum*.*

*G. Morton Journal of Contemporary Brachytherapy 2014/volume 6/number 3*

Where homogenous coverage of the gland is required then catheters should be placed with no greater than 1 cm intervals between applicators. It is also important to remember that applicators placed closer than this to the urethra and rectum may not be able to contribute maximally to the dose distribution due to the OAR constraint. Peripheral coverage is the most important so it is vital to have a ring of catheters around the edge of the peripheral zones, with a distance of about 3 mm from the prostate CTV border. It is advantageous to start to implant with the anterior catheters. This allows early checking of interference with the pubic arch so that adjustments can be made to set up early on to overcome this. It also minimizes problems from the ultrasound shadowing effect behind the implant needles which decreases the image quality. It is also important to scroll up and down the ultrasound images during implantation to ensure there is not only good cover at the center of the gland but also at the base and apex where the volume tapers and may require a second inner ring of applicators to deliver an adequate dose to this region.

*GEC/ESTRO recommendations on high dose rate afterloading brachytherapy for localized prostate cancer: an update. P. Hoskin Radiother Oncol 2013;107: 325-332*

**Appendix B**

**Appendix B-1. Basic 3D Ultrasound Image Acquisition Steps**


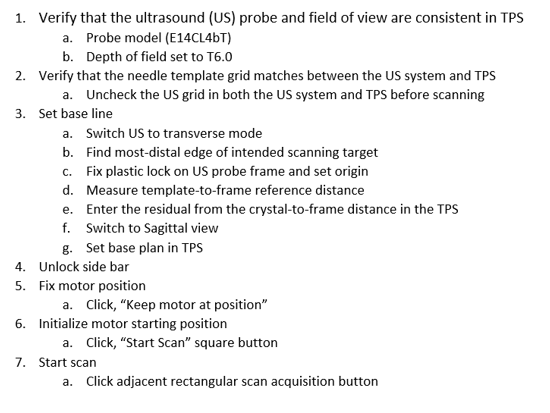


**Appendix B-2. Basic Operation of Stepper and Stabilizer**


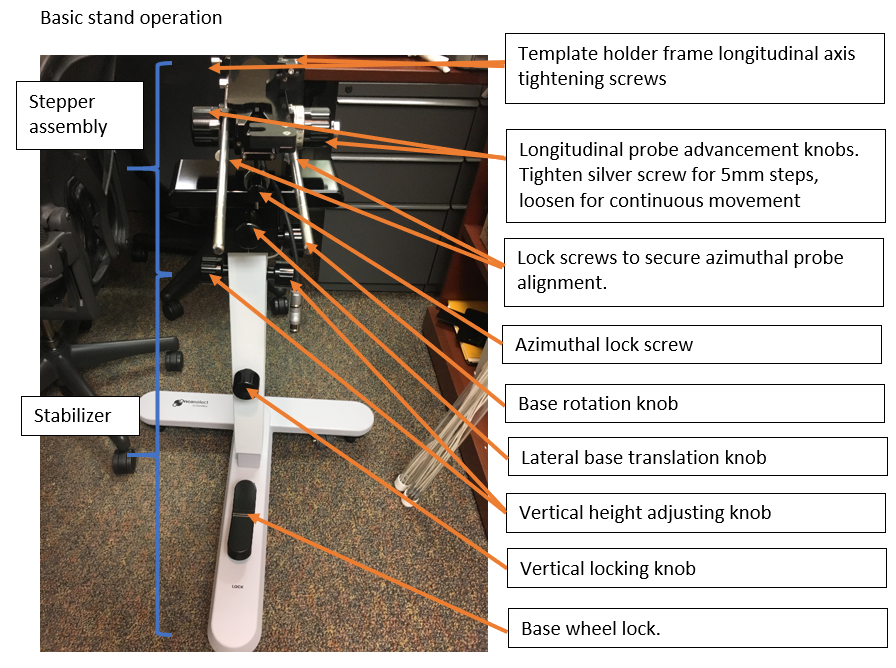


**Appendix C**

**Appendix C-1 Simple overall relationship of each length and how to OCP calculates free length**


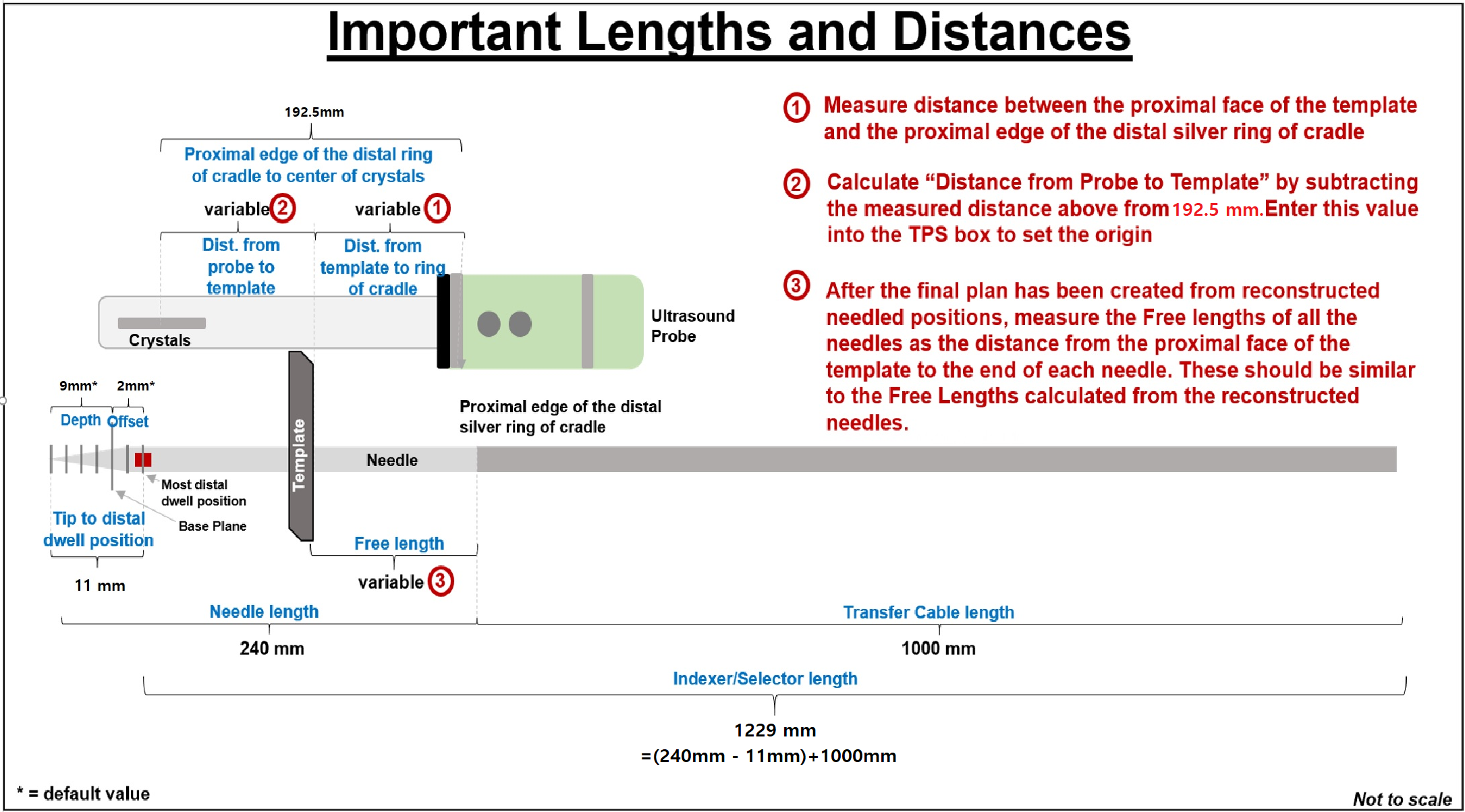


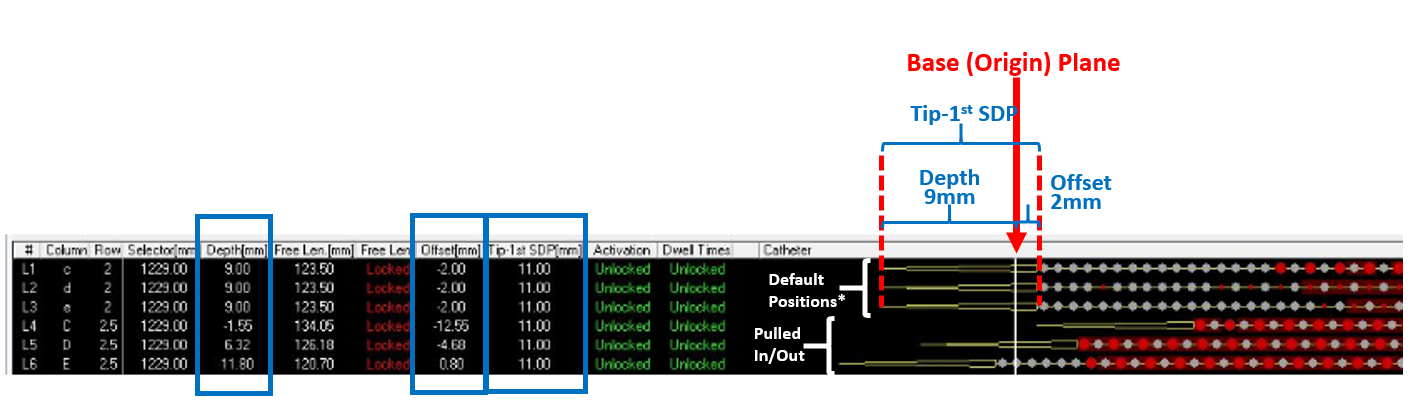


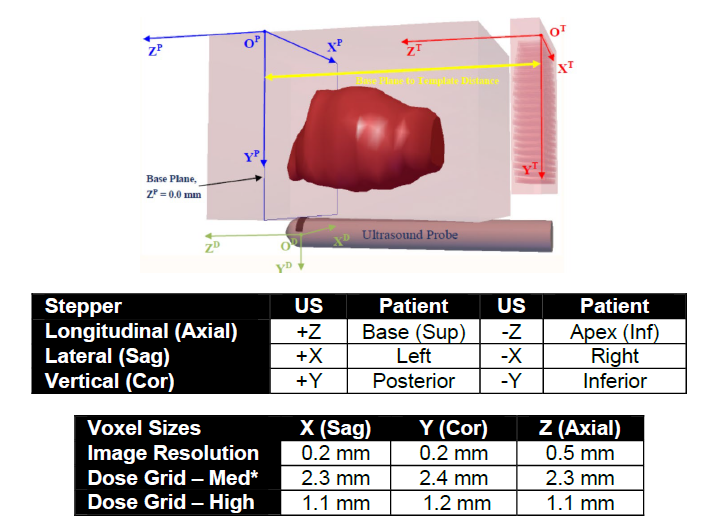


**Appendix C-2 Detailed free length calculation in OCP for each needle insertion**


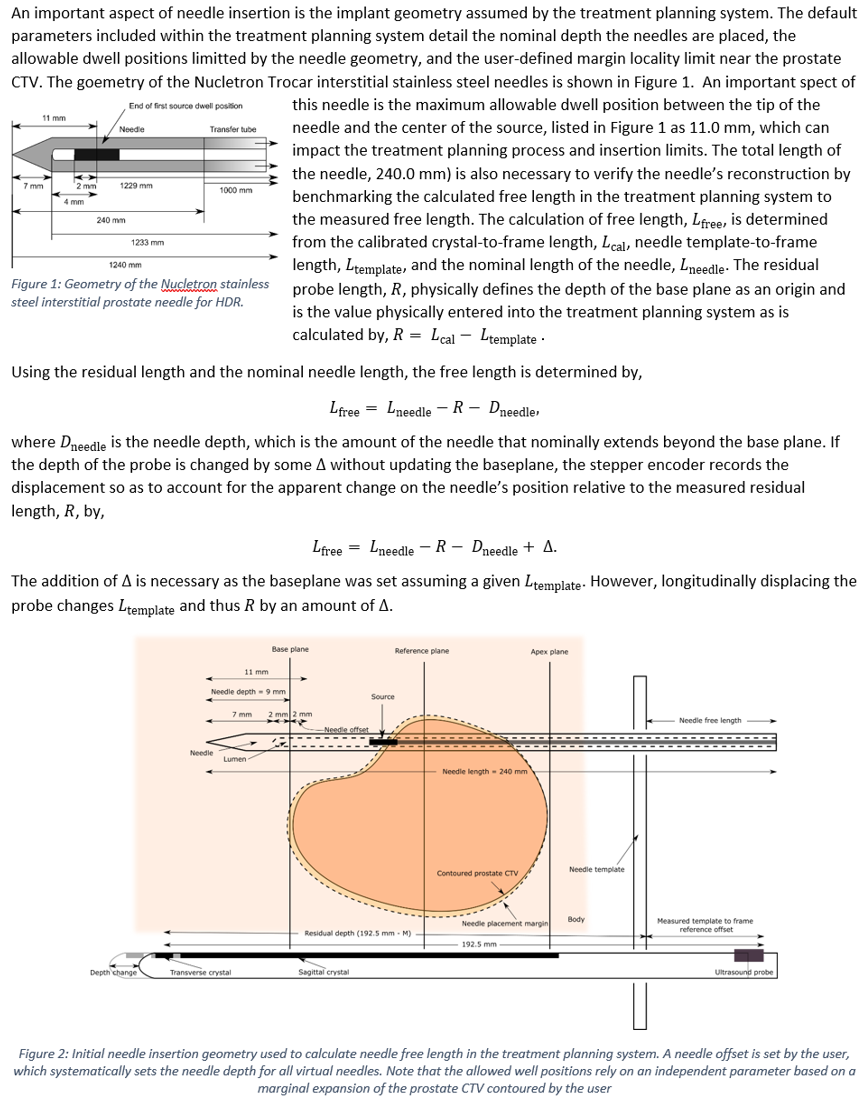


**Appendix D. RT Chart Checklist for Prostate HDR Brachytherapy**

**
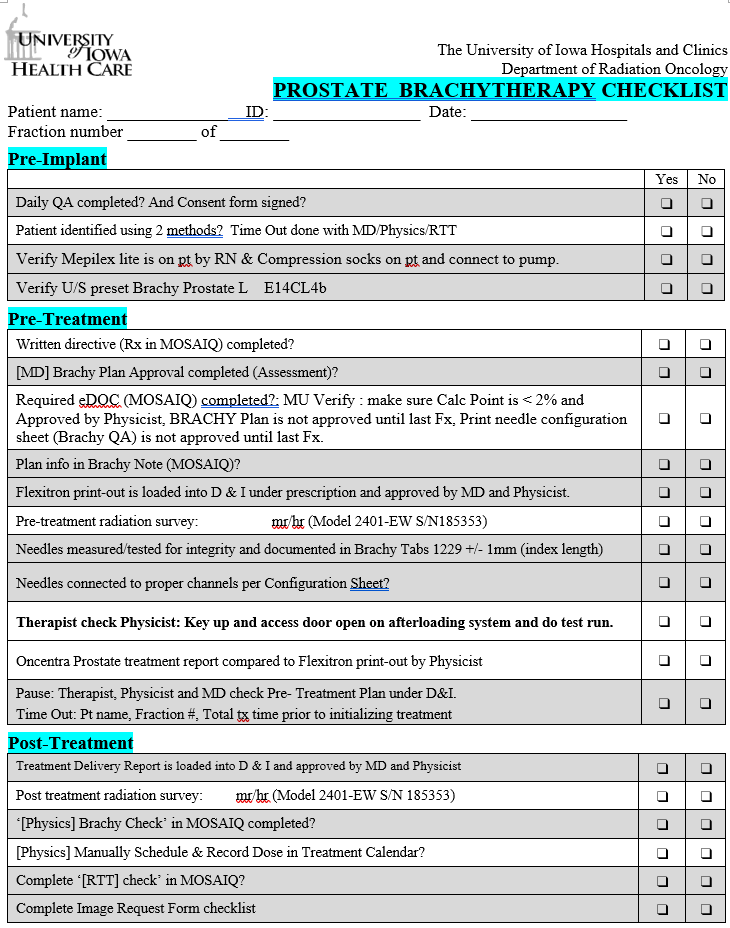
**

**Appendix E. UIHC Prostate HDR – Planning Physicist Checklist**


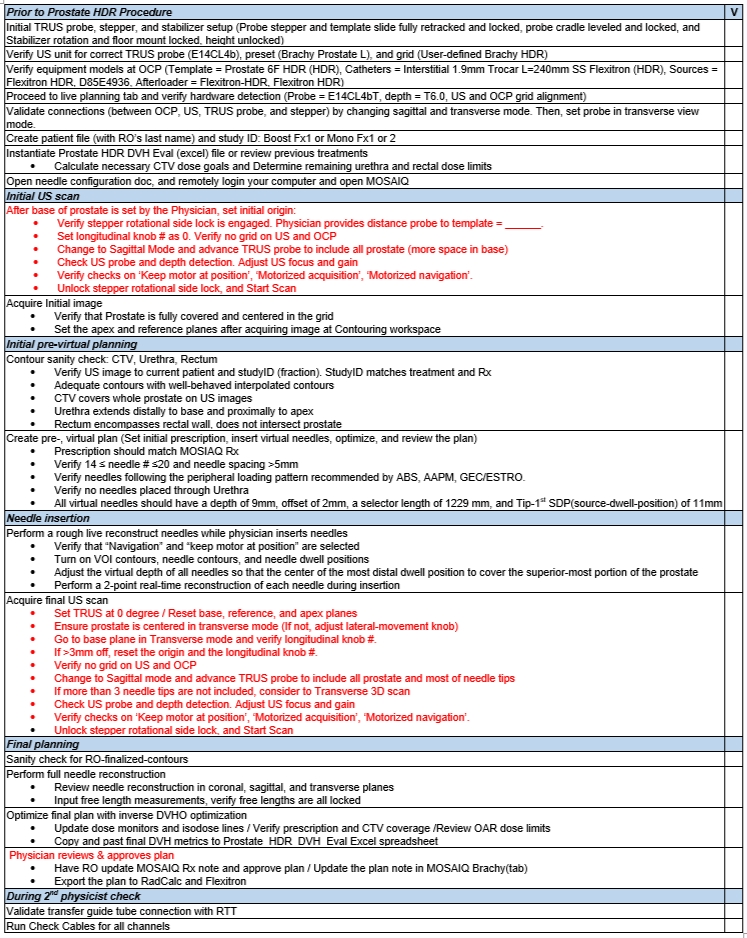


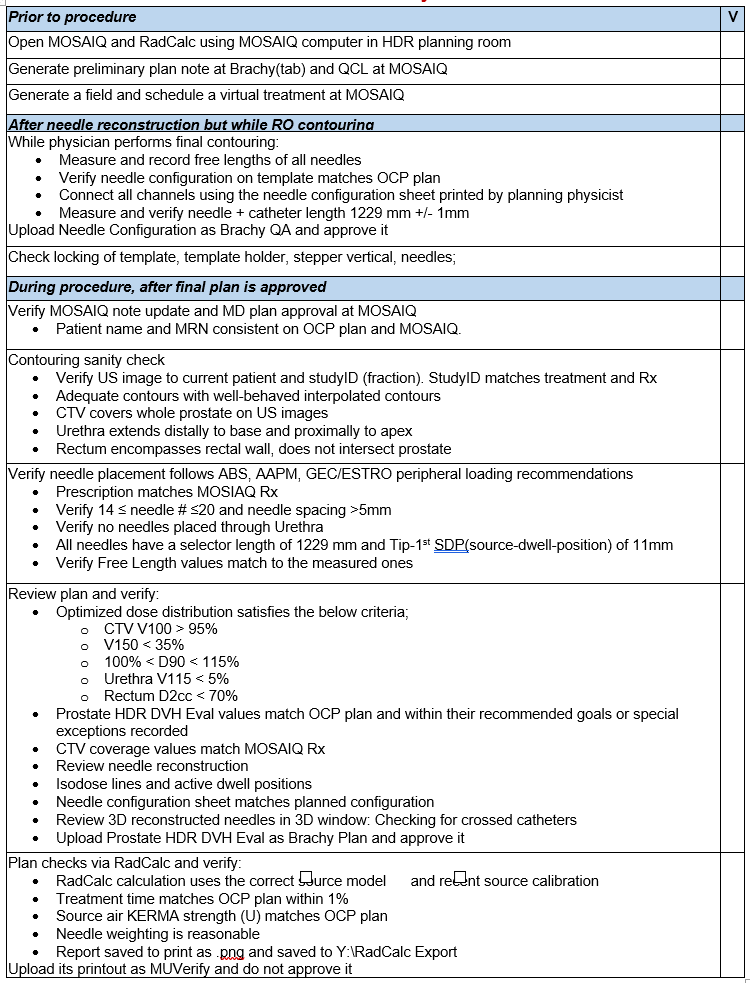
**Appendix F. UIHC HDR Prostate – 2nd Physicist Checklist**

**Appendix G. UIHC Prostate HDR Equipment Checklist**

**
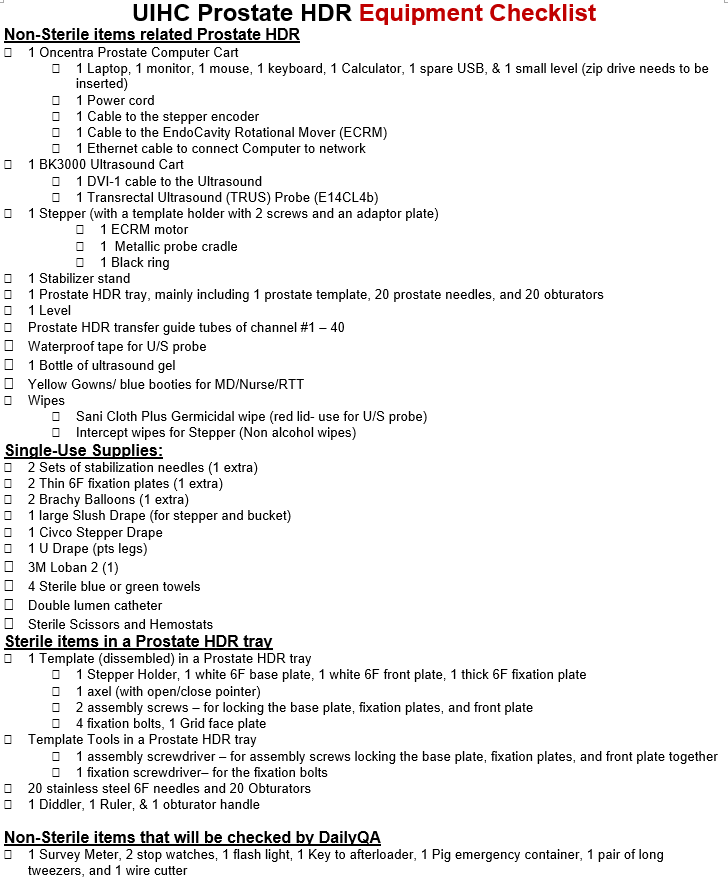
**

**Appendix H - Sterilization and Cleaning Instruction**

# **Equipment and necessary method**

| **Item** | **Method** | **Instructions Document** | **Section/Page** |
| --- | --- | --- | --- |
| Template and accessories | Sterilize | Cleaning&Sterilization | Section 13  S:\OncShare\PHYSICS\Elekta Prostate HDR\Others\Manual IFU Catalogue Quotes\IFU Manuals\IFU Template |
| Obturators and Needles | Sterilize | Cleaning&Sterilization | Section 13 and 14  S:\OncShare\PHYSICS\Elekta Prostate HDR\Others\Manual IFU Catalogue Quotes\IFU Manuals\IFU_Needles_Transfer Tubes |
| Rulers | Sterilize | N/A | N/A |
| Diddler | Sterilize | N/A (can be autoclaved) | N/A |
| Ultrasound | High-Level Disinfectant | N/A | N/A |
| Stepper, cradle, ring, motor | Clean | Cleaning&Sterilization - Stepper.pdf | Section 5.1.1  S:\OncShare\PHYSICS\Elekta Prostate HDR\Others\Manual IFU Catalogue Quotes\IFU Manuals\IFU Stepper |
| Transfer tubes | Clean | N/A | N/A |

# **Requiring Cleaning Only**

# **Equipment List**

1. Oncentra Prostate Computer Cart, 2 cables for stepper encoder & motor/ECRM), and accessories
2. BK3000 Ultrasound Cart and 1 cable (DVI)
3. Large Level
4. Transfer guide tubes
5. Stepper:
   1. 1 black probe holder ring (Figure 1)
   2. 1 stepper head (probe and template holder with the encoder knobs) (Figure 1)
   3. 1 silver probe cradle (Figure 2)
   4. 1 Motor/Endo-Cavity Rotational Mover (ECRM) (Figure 2)
   5. 1 stabilizer (stand)
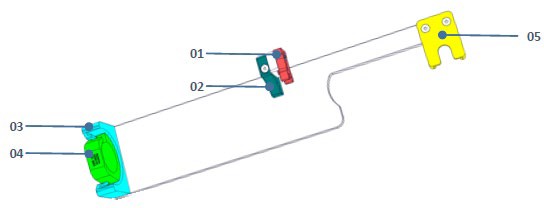

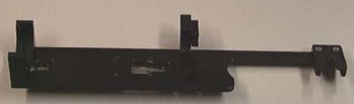

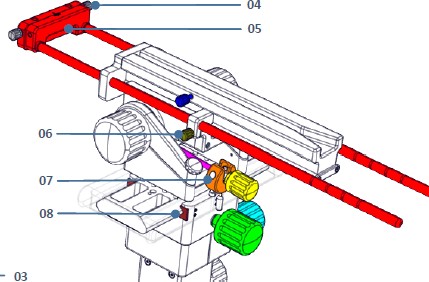

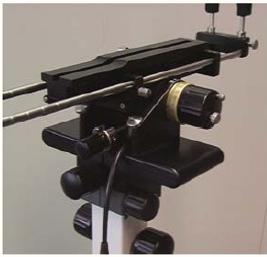


**C**

**A**

**B**

**Figure 1**. The stepper head refers the stepper assembly (A and B) where a template support is the red part (B). BK adapter plate (C) is a part of stepper head where the green part in the small diagram of Left represents ‘black probe holder ring’.


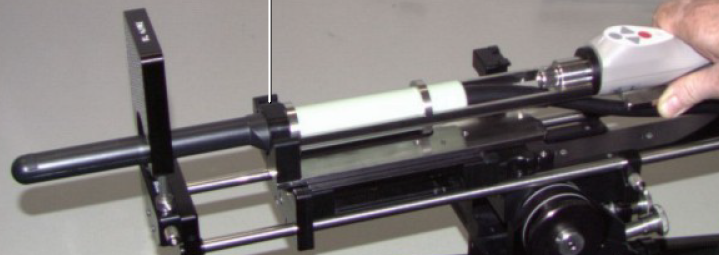


Silver probe cradle

Motor/ECRM

Black probe holder ring

**Figure 2**. The figure presents a black probe holder ring, a silver probe cradle, and a motor or Endo-Cavity Rotational Mover (ECRM) in a stepper head.

# **Cleaning instructions at HDR suite**

1. **Transfer tubes:**
2. Physics should wipe down the transfer tubes using the non-alcohol intercept wipes before putting them back in the wall containers
3. **Cables of Oncentra Prostate TPS Cart:**
4. Physics disconnect two cables connected to stepper head (Figure 1 A) and the US unit.
5. Physics should wipe down two cables of the stepper encoder and the ECRM using the non-alcohol intercept wipes before putting the Oncentra Prostate Cart back in the HDR planning room
6. **Stepper Head:**
7. RTT remove the template support arm (Figure 1 B, the red part), a silver cradle (Figure 2) and a black probe holding ring (Figure 2) from the stepper head.
8. RTT remove the template locking screws (two of them) from the support arm.
9. RTT remove the BK adapter plate (Figure 1 C).
10. RTT disassemble the silver cradle from the motor / ECRM (Figure 2) and the black probe holder ring (Figure 2) from the cradle.
11. RTT clean all parts with the non-alcohol intercept wipes
12. RTT use the brushes to thoroughly clean the template locking screw holes (this is where blood can easily get stuck).
13. RTT wipe down and clean a silver cradle and a black probe holder ring using the non-alcohol intercept wipes.
14. RTT be sure to get all of the blood off from the rest of stepper head (Figure 1 and 3) using the non-alcohol intercept wipes.
15. You may also need to wipe down with the black wipes or a paper towel afterward to remove any white residue that the bleach wipes might leave.
16. **Motor / ECRM:**
17. RTT should thoroughly wipe down the motor / ECRM (Figure 2) carefully using the black wipes (should not be that dirty)
18. RTT wipe down again the motor / ECRM with the black wipe if needed (this should not be that dirty)
19. RTT use either the non-alcohol intercept wipes and wipe outside of the encoder (item 1), the whole cable (item 2) and connector (item 3), and magnetic strip (item 4) (Figure 3)
20. RTT do not place the encoder connector in any solvent or liquid (wipe off only)


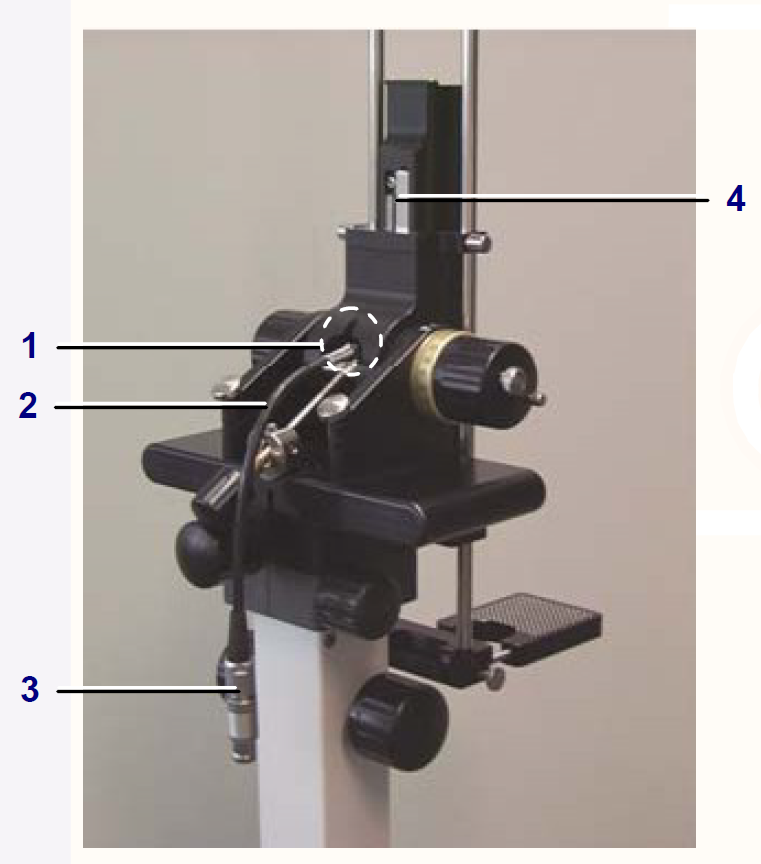


**Figure 3**. The figure presents the encoder (item 1), the whole cable (item 2), and connector (item 3), and magnetic strip (item 4) in the stepper head.

1. **Needle Template:**
2. RTT dissemble the template from the stepper head.
3. RN put the yellow caps on the needles.
4. RN run tap water on the template and needles.
5. RN takes the dirty biomed bin to the Rad Onc Soiled utility room


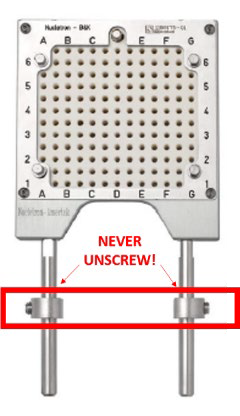


**Figure 3**. BK template. RN never unscrew the vertical adjustment screws on the legs! (No screwdriver for this screws is available in the prostate HDR tray).

1. **Prostate HDR Tray:**
2. RN make sure all the items in the prostate HDR tray
3. 1 metal ruler
4. 1 diddler
5. 1 obturator handle
6. 1 Stepper Holder Assembly (Nucletron/Elekta #189.095 (B-K))
7. 1 white 6F base plate (Nucletron/Elekta #189.158)
8. 1 white 6F front plate (Nucletron/Elekta #189.161 (6F))
9. 1 thin 6F fixation plate (Nucletron/Elekta #189.178 (6F)): disposable
10. 1 thick 6F fixation plate (Nucletron/Elekta #189.166)
11. 1 axel (with open/close pointer) (Nucletron/Elekta #189.167)
12. 2 assembly screws (Nucletron/Elekta #189.170)
13. 4 fixation bolts (Nucletron/Elekta #189.168)
14. 1 Grid face plate [note: Labels are A-G and 1-6] (Nucletron/Elekta #189.175 (B&K))
15. 1 assembly screwdriver – for assembly screws (Nucletron/Elekta #189.205)
16. 1 fixation screwdriver – for the fixation bolts and locking axel (Nucletron/Elekta #189.173)
17. RN count and make sure there are 20 used and unused needles.
18. RN count and make sure there are 20 used and unused obturators.
19. RN place them in a dirty biomed bin and soak instruments in Hydrogen Peroxide per instructions.
20. **Transrectal Ultrasound (TRUS) Probe:**
21. RTT should wipe down the US cable (DVI) using Non-alcohol Intercept Wipes (blue and white container) connecting to the Oncentra Prostate cart, along with cleaning the BK3000 US unit
22. RTT disassemble the TRUS probe from the stepper head and put it in a disposable tray.
23. RTT cleans the TRUS probe with the Sani Cloth Plus Germicidal Wipe (Red Top)
24. RTT takes the disposable tray of a TRUS probe in the Rad Onc Soiled room.

# **Cleaning instructions at Rad Onc Soiled room**

1. **Needle Template:**
2. Disassembles each part of the template.
3. Never unscrew the vertical adjustment screws on the legs: This is very important – it will mess up the template position calibration!
4. Throw away a disposable metal plate (thin one).
5. Puts the template in a dirty biomed bin, soak instruments in Hyrogen Peroxide per instructions.
6. Remove visible bioburden utilizing Intercept wipes (UIHC approved) and/or alternate product supplied/approved by UIHC
7. Dissemble equipment that is to be sent to CSS for sterilization
8. Uses the brushes to thoroughly clean the template (this is where blood can easily get stuck).
9. For the template:
   1. The 3 x 100 mm (part number 189.041) brush is for the needle holes
   2. The 6.5 x 100 mm (part number 189.043) brush is for the screw holes
10. Spray equipment with “forever wet” following dissembling
11. When finished, MA process the prostate HDR tray sterilization through CSS.
12. **Transrectal Ultrasound (TRUS) Probe:**
13. MA remove visible bioburden utilizing Intercept wipes (UIHC approved) and/or alternate product supplied/approved by UIHC
14. MA reassemble/inventory equipment that is to be sent to FERC for high level disinfection
